# QSAR-Guided and Fragment-Based Drug Design of Monoterpenoid Inhibitors Targeting Ebola Virus Glycoprotein

**DOI:** 10.3390/ijms27072987

**Published:** 2026-03-25

**Authors:** Nouhaila Ait Lahcen, Wissal Liman, Saad Zekri, Adnane Ait Lahcen, Ashwag S. Alanazi, Mohammed M. Alanazi, Christelle Delaite, Mohamed Maatallah, Driss Cherqaoui

**Affiliations:** 1 Molecular Chemistry Laboratory, Faculty of Sciences Semlalia, Cadi Ayyad University, UCA, Marrakech 40000, Morocco; s.zekri.ced@uca.ac.ma (S.Z.); adnane.aitlahcen.ced@uca.ac.ma (A.A.L.); cherqaoui@uca.ma (D.C.); 2 Bioinformatics Laboratory, College of Computing, University Mohammed VI Polytechnic, Benguerir 43150, Morocco; wissal.liman@um6p.ma; 3 Department of Pharmaceutical Sciences, College of Pharmacy, Princess Nourah bint Abdulrahman University, Riyadh 11671, Saudi Arabia; asalanzi@pnu.edu.sa; 4 Department of Pharmaceutical Chemistry, College of Pharmacy, King Saud University, Riyadh 11451, Saudi Arabia; mmalanazi@ksu.edu.sa; 5 Laboratoire de Photochimie et d’Ingénierie Macromoléculaires (LPIM), Ecole Nationale Supérieure de Chimie de Mulhouse, Université de Haute-Alsace, 68100 Mulhouse, France; christelle.delaite@uha.fr; 6 Sustainable Materials Research Center (SUSMAT-RC), University of Mohammed VI Polytechnic, Benguerir 43150, Morocco

**Keywords:** Ebola virus, EBOV-GP, monoterpenoids, QSAR, fragment-based drug design, molecular docking, MD simulation, MM/PBSA

## Abstract

Ebola virus disease remains one of the most serious viral infections with no approved small-molecule treatments. The Ebola virus glycoprotein (EBOV-GP), which enables the virus’s entry to host cells, is a promising target for drug discovery. In this study, a multistage computer-aided drug discovery approach was used to identify new specific EBOV-GP inhibitors. A reliable QSAR model was built using 55 terpenoid derivatives. This model was able to predict the activity of newly designed compounds with good accuracy and validated statistical metrics ($R_{tr}^{2}\;$= 0.70; $R_{ext}^{2}$ = 0.73). It was subsequently applied to screen over 15,500 newly generated compounds from three lead molecules by fragment-based design tools. Predicted activity, binding affinity toward EBOV-GP, and good ADMET drug-like properties prioritized the eleven most promising hits. Through 150 ns molecular dynamics simulations, these compounds remained stable in the EBOV-GP binding site. Further binding free energy analysis (MM/PBSA) showed strong binding affinities, especially for the compounds **L-60**, **L-832**, **M-1618**, and **L-1366**. This study showed how combining QSAR, fragment-based design, docking, ADMET, and molecular dynamics could help in identifying potent and safe small molecules against the EBOV-GP. The top compounds are ready for further experimental and in vitro biological testing.

## 1. Introduction

Emerging infectious diseases are recurring as a reminder of how quickly biological threats can challenge global health systems. Among them, Ebola virus (EBOV), a member of the *Filoviridae* family, is the etiological agent of Ebola virus disease (EVD) [1]. It is a highly infectious and frequently lethal hemorrhagic fever with historical fatality rates reaching up to 90% [2]. The 2014–2016 West African epidemic and subsequent outbreaks of 2021, 2022, and 2023 have reinforced the urgency of implementing robust therapeutic countermeasures targeting this virus [3]. Despite the development of monoclonal antibody cocktails and vaccine candidates (Inmazeb and Ebanga), no small-molecule antiviral has yet received approved status for the treatment of EVD [1,4,5,6].

This persistent gap has redirected attention to the molecular machinery of EBOV and its structural determinants of host-cell entry, which are becoming increasingly important to current therapeutic research. A deeper understanding of the molecular steps that underline EBOV entry into host cells is therefore essential for the rational design of effective antiviral strategies. This is because entry inhibition delays viral propagation at an early stage, minimizing the virus’s ability to acquire drug resistance during a later step of virus spread.

In this context, the Ebola virus glycoprotein (EBOV-GP) has emerged as a particularly interesting area of research because it plays a central role in the viral life cycle. EBOV-GP is the primary mediator of viral attachment, fusion, and endosomal entry into host cells [7,8]. Although mapping stable binding sites has been challenging because of EBOV-GP’s notable conformational flexibility, particularly during endosomal trafficking transitions and membrane fusion, these same structural dynamics highlights its potential as a druggable target [9]. Therefore, interfering with EBOV-GP activity during the entry stage may prevent replication at its most strategic point, providing a defensive opportunity for the creation of small-molecule treatments.

To better benefit from this opportunity efficiently, a more rapid development pathway involves the integration of in silico approaches, which allow high-throughput molecular screening, mechanistic modeling, and rational design with minimization of both the cost and time associated with traditional methods [10,11]. Within this computational arsenal, Quantitative Structure–Activity Relationship (QSAR) modeling and fragment-based drug design (FBDD) represent an excellent complementary paradigm. QSAR uses statistical learning to model the relationship between molecular descriptors and biological activity; it is capable of predicting the biological activities of newly designed compounds before their experimental synthesis [12]. FBDD explores chemical space by iteratively expanding or replacing pharmacophoric fragments prioritizing strong-binding candidates [13].

This study employs a multistage computational framework to identify potent EBOV- GP inhibitors by integrating QSAR modeling and FBDD. The workflow begins with the development of a QSAR model to guide the evaluation of a virtual library of fragment analogs derived from terpenoid scaffolds. Designed compounds are subsequently prioritized using QSAR-based filtration, molecular docking scoring against a modeled EBOV-GP receptor, and ADMET profiling. The stability and conformational behavior of top candidates complexed with EBOV-GP are further examined using molecular dynamics simulations and the MM/PBSA approach. Likewise, Density Functional Theory (DFT) calculations are employed as a quantum chemical validation and refinement step, complementing the preceding approaches by providing atomic-level insight into the global reactivity and electronic properties of the selected compounds. For antiviral intervention against EBOV, this pipeline offers a rational path toward discovering new EBOV-GP inhibitors with favorable binding and pharmacokinetic properties, which accelerate the early stages of drug design, and lays the groundwork for further experimental exploration.

## 2. Results and Discussion

### 2.1. QSAR Model Performance and Predictivity

The GA-MLR best model was constructed using four molecular descriptors as indicated by the Q^2^ versus R^2^ visualization, which stabilized upon fixing the number of descriptors at four. They were selected from a pool of 721 features based on their statistical relevance and contribution to model performance and are VE3sign_X, VE3sign_Dt, SpMAD_AEA(dm), and MaxssCH2, which capture key topological and electronic features of the compounds. Detailed definitions and physicochemical interpretations of these descriptors are provided in Table S2. The resulting Equation (1) and the statistical parameters of this model are shown below:

pIC_50_ = 16.3777 + 0.0991(VE3sign_X) − 0.3971(VE3sign_Dt) − 16.5401(SpMAD_AEA(dm)) + 10.583(MaxssCH2) (1)

N_Tr_ = 45; R^2^_Tr_ = 0.70; MAE_Tr_ = 0.3318
N_ext_ = 10; R^2^_ext_ = 0.73; MAE_ext_ = 0.2438
Q^2^_F1_ = 0.7326; Q^2^_F2_ = 0. 7169; Q^2^_F3_ = 0.7406
CCC_ext_ = 0.8500; F = 23.0447; s = 0.4156

**Figure 1:**
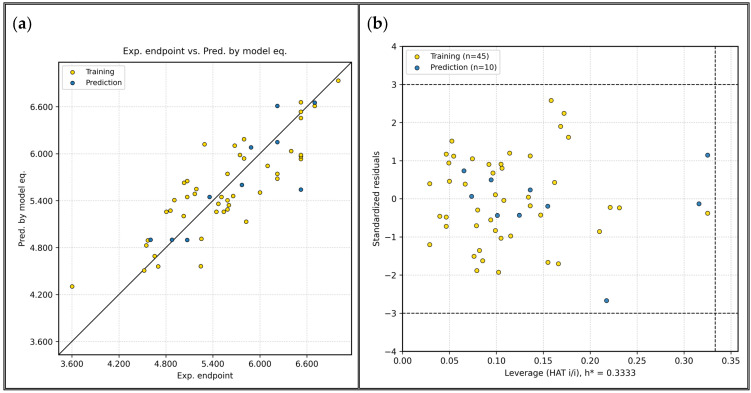
(**a**) Plot of experimental vs. predicted pIC_50_ values. (**b**) Williams plot for applicability domain assessment.

This model demonstrated excellent statistical robustness and predictive capability. Figure 1a shows strong correlation between the experimental pIC_50_ values and those predicted by the GA-MLR model and highlights the predictive significance of the selected molecular descriptors. The training set yielded an R^2^ of 0.70 while the external validation has an R^2^_ext_ of 0.73 and Q^2^_F1_ = 0.7326, indicating a strong correlation between molecular descriptors and pIC_50_. The model satisfied Tropsha’s necessary criteria [14] for acceptable predictive QSAR models, passed Y-randomization tests, and affirmed its reliability. The AD was visualized via Williams plot (Figure 1b). It highlights a structurally diverse but organized chemical space. Compounds exceeding the leverage threshold (h*) of 0.333 were flagged but retained for prospective analysis due to mechanistic interest. For this model, all molecules fall within the applicability domain. The Williams plot showed that no compound exceeded the leverage threshold (h*) or the ±3 standardized residual limits, indicating the absence of outliers and influential observations. This result confirms that the descriptor space was adequately sampled and that no individual molecule exerted excessive influence on the model. Thus, all compounds fall within the defined applicability domain, supporting the robustness of the model and the reliability of predictions for new analogs within this chemical space.

### 2.2. Fragment Library Expansion and QSAR-Guided Screening

Fragment-based design using the FragGrow and FragRep platforms generated a large and structurally diverse analog library derived from the three potent scaffolds presenting the highest pIC_50_ (**7c**, **12c**, and **4d**). All of these compounds were screened using the validated GA-MLR QSAR model to estimate their inhibition activity against EBOV-GP. A threshold of pIC_50_ ≥ 6.5 was applied to prioritize candidates with predicted biological relevance. This filtering step reduced the library size while preserving structural variety suitable for next analyses.

The AD was evaluated using a Williams plot based on the model leverage values. Out of the 15,544 newly generated compounds, 12,556 molecules (80.8%) were found to fall within the model’s AD (Figure S1). This high proportion indicates that most of the designed compounds remain within the descriptor space defined by the training set, supporting the reliability of the model predictions. Notably, this outcome is consistent with the design strategy adopted in this study, in which the core scaffold was preserved while structural diversification was introduced through fragment-growing and fragment-replacement strategies on peripheral substituents and selected linker regions. Consequently, the generated library maintains structural similarity with the original chemical space while allowing exploration of optimized analogs with potentially enhanced activity.

FragGrow generated analogs through iterative fragment expansion, resulting in structure-specific sets reflecting the distinct steric environments of the starting molecules. FragRep, on the other hand, produced a greater number of derivatives through substitution, a comparatively higher proportion of which met the QSAR selection criteria. This result confirms the hypothesis that structures preserving modifications often maintain favorable electronic and steric features more than growth-based expansions. After this QSAR-based filtration, 296 FragGrow-derived and 912 FragRep-derived compounds were preserved, all of which fall within the AD of the developed QSAR model. These analogs were advanced to molecular docking analyses to further assess their interaction with the EBOV-GP binding site.

### 2.3. Molecular Docking Insights and ADMET Profiles

Comprehensive loop reconstruction was executed to recover the full conformational landscape of EBOV-GP. The crystal structure (PDB 7M2D) was first superposed on a higher-resolution homolog, after the missing residues were rebuilt with ProMod3. Quality assessment showed GMQE and QMEAN scores of 0.72 and 0.69, respectively, confirming stereochemical accuracy and global reliability. A PROCHECK Ramachandran analysis (Table 1 and Figure S2) yielded an overall quality factor of 88.2; 90.2% of backbone dihedrals resided in the most favored regions, 9.2% in additionally allowed regions, none in generously allowed regions, and only 0.6% in disallowed regions. The associated Z-scores for first-generation packing quality (−1.69) and plot appearance (−1.18) fell within the acceptable range for high-quality models, indicating that the rebuilt loops introduced neither steric clashes nor backbone strain. These metrics validate the model’s suitability for later docking studies and extended MD simulations directed toward EBOV-GP inhibitor design.

The reliability of the molecular docking protocol was first assessed via a re-docking study of the native ligand, YPS, in the binding site of the EBOV-GP. The re-docked pose was then aligned with the native conformation. The resulting RMSD between the docked and co-crystalized structures was 0.7472 Å (Figure S3), which falls well below the accepted threshold of 2.0 Å. This low RMSD value confirms the accuracy and robustness of the docking methodology to be employed in this study.

The 1208 compounds filtered using the validated QSAR model were subsequently subjected to molecular docking-based virtual screening against the EBOV-GP binding site (Files S1–S6). The reference compounds, toremifene and the three lead compounds, were first docked. Toremifene exhibited a binding affinity of −6.9 kcal/mol, while the compounds **7c**, **12c**, and **4d** showed moderately improved affinities of −7.2, −7.7, and −7.6 kcal/mol, respectively. Based on the average binding affinity of the three leads, a cutoff value of −7.5 kcal/mol was applied to prioritize candidates exhibiting stronger predicted affinities as well as binding interactions. Following this criterion, 37 molecules from the FragGrow dataset were retained: 21 originating from lead **7c**, 13 from lead **12c**, and 3 from lead **4d**. Meanwhile, from the FragRep dataset, 146 molecules met the cutoff. This includes 52 from **7c**, 46 from **12c**, and 48 from **4d**.

To further evaluate the safety profiles of these candidates, an ADMET filter was applied to select the most promising and non-toxic compounds. From the FragGrow dataset, four molecules passed the ADMET criteria: two derived from **7c** and two from **12c**. Similarly, seven molecules from the FragRep dataset were selected: two from **7c**, two from **12c**, and three from **4d**. The eleven compounds showed pharmacokinetic and physicochemical profiles compatible with oral administration and acceptable safety margins (Table 2). All candidates complied with Lipinski’s Rule of Five, passed the Golden Triangle screen, and were PAINS-negative. Molecular weights were below 500 Da and the calculated logP values ranged from 1.43 to 4.76, which is adequate for membrane permeability without excessive lipophilicity. Predicted aqueous solubility (logS from −2.44 to −5.30) remained within the range considered suitable for oral dosage forms, with **L-1542**, **L-1366**, and **L-874** displaying the best solubility. Caco-2 permeability exceeded the 1.0 threshold for every compound, and the human intestinal absorption (HIA) scores were >90%. **L-1542** and **M-1435** approached complete absorption. Blood–brain barrier penetration was modest. The highest logBB values (0.279) were noted for **L-1366** and **L-874**, but none of the molecules showed a propensity for excessive central nervous system exposure. **M-1205**, **M-1435**, and **L-60** were predicted to inhibit CYP2C19 and/or CYP2C9, a liability attributable to their bulky hydrophobic scaffolds and heteroatom content (N, O, and S). Although this raises the potential for drug–drug interactions, comparable profiles are common among approved antivirals and other lipophilic drugs and can be addressed through optimization and in vitro CYP assays. Predicted systemic clearance values lay between 1.04 and 1.35 log(mL·min^−1^·kg^−1^), only **M-1435** fell marginally below the typical relevance threshold. Synthetic accessibility scores (4.74–5.38) indicate routes of moderate complexity, feasible for routine medicinal chemistry. The moderate increase in **L-60** SA score reflects the structural complexity of the molecule, including its bicyclic core, stereochemical features, and multifunctional architecture, which may necessitate multistep synthesis. Toxicity risk assessments were favorable as all evaluated molecules were predicted to be negative for AMES mutagenicity, hepatotoxicity, genotoxicity and carcinogenicity. However, compound **L-1542** exhibited a single alert related to the genotoxicity–carcinogenicity–mutagenicity assessment, representing a precautionary flag rather than a confirmed toxic liability, whereas all remaining compounds showed no alerts.

In summary, the eleven ligands combine favorable pharmacokinetic parameters, manageable synthetic routes, and an absence of critical toxicity warnings, supporting their progression as optimized EBOV-GP inhibitors.

Regarding their binding, all newly designed compounds from both approaches demonstrate enhanced binding with affinities ranging from −7.6 to −9.0 kcal/mol as summarized in Table 3. For example, **L-3796** exhibited the strongest predicted interaction (−9.0 kcal/mol), followed by **L-60** (−8.6 kcal/mol) and **M-1074** (−8.2 kcal/mol). These results highlight the success of the fragment-based design strategy for optimizing ligand binding.

**Figure 2:**
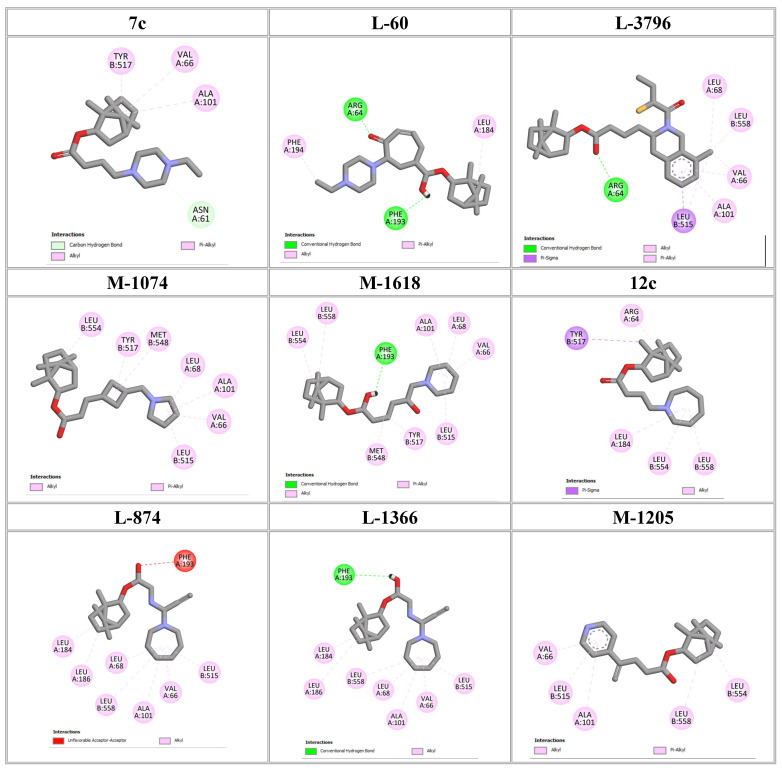

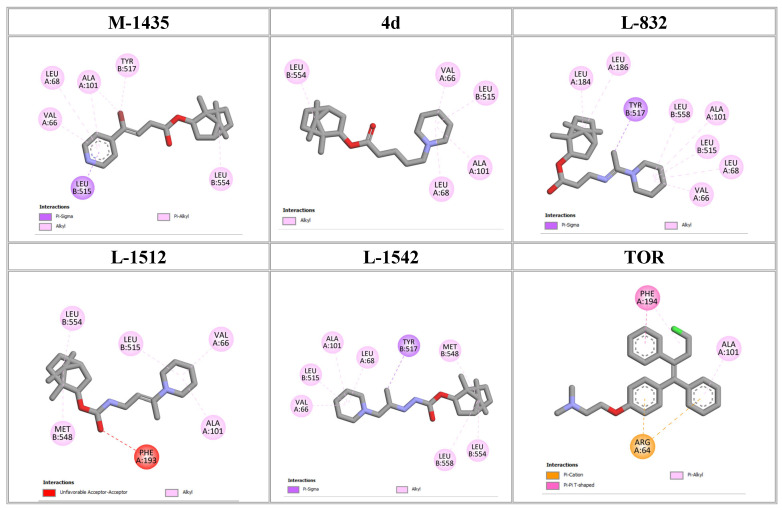
2D interaction of newly designed EBOV-GP inhibitors, the three leads compounds, and TOR within the active site of EBOV-GP.

To elucidate how the eleven newly designed ligands bind in the EBOV-GP pocket, their docking poses were examined in detail (Figure 2), focusing on hydrogen bonding, hydrophobic contacts (alkyl, π-alkyl, and π-sigma), and, where present, electrostatic contributions. **L-60** was anchored by four classical hydrogen bonds to ARG64 and PHE193 while reinforcing the fit with alkyl contacts to LEU184 and PHE194, an effective blend of polar and nonpolar interactions. **L-3796** shows the richest network with the presence of one hydrogen bond to ARG64 and seven hydrophobic contacts with VAL66, LEU68, ALA101, LEU515, and LEU558, indicating well-dispersed placement in the cavity. **M-1074** relies only on hydrophobic forces, stacking against VAL66, LEU68, ALA101, MET548, LEU544, LEU515, and TYR517, a signature of deep burial in the nonpolar core. Likewise, **M-1618** combines a single hydrogen bond to PHE193 with extensive van der Waals contacts to VAL66, LEU68, ALA101, MET548, and TYR517, yielding robust steric stabilization. The compounds **L-874**, **L-1366**, and **L-832** exhibited comparable hydrophobic interaction arrangements within the binding site with the engagement of conserved residues including VAL66, LEU68, ALA101, LEU184, LEU186, LEU515, and LEU558. The key differences among these ligands were observed in their interactions with specific aromatic residues. **L-874** displayed an unfavorable acceptor–acceptor contact with PHE193, whereas **L-1366** formed a stabilizing hydrogen bond with this residue. In addition, **L-832** established a π-σ interaction with TYR517, further contributing to its binding stabilization. The compounds **M-1205**, **L-1435**, **L-1512**, and **L-1542** exhibited predominantly hydrophobic binding profiles within the binding site. **M-1205** engaged in hydrophobic interactions with VAL66, ALA101, LEU515, LEU554, and LEU558. **L-1435** similarly formed hydrophobic contacts with VAL66, LEU68, ALA101, LEU517, and LEU554, in addition to a π-σ interaction with LEU515. **L-1512** maintained hydrophobic interactions with VAL66, ALA101, LEU515, MET548, and LEU554, but also exhibited an unfavorable acceptor–acceptor interaction with PHE193. In contrast, **L-1542** formed hydrophobic contacts with VAL66, ALA101, LEU515, LEU554, LEU558, and MET548 and additionally established a π-σ interaction with TYR517.

The original leads are less interactive; **7c** forms a single carbon-type hydrogen bond to ASN61 plus sparse hydrophobic links; **12c** relies on five hydrophobic contacts with no hydrogen bonding; and **4d** engages only through alkyl interactions with VAL66, LEU68, and LEU554. The reference ligand toremifene displays a unique profile dominated by π-cation and π-π contacts to ARG64 and PHE194. Overall, the new series outperforms the earlier leads by forging denser, strategically placed contacts, especially with VAL66, ALA101, MET548, and TYR517, while retaining or improving hydrogen-bond capacity [15]. This expanded interaction repertoire supports their candidacy as refined EBOV-GP inhibitors. (A full interaction description is provided in Table S3).

Reported small-molecule series, such as diarylsulfides and diarylamines, bind hydrophobic cavities at the GP1-GP2 interface or near the receptor-binding region, thereby preventing the conformational rearrangements required for fusion or disrupting the interaction with the host receptor Niemann–Pick C1 (NPC1) [16]. Structural and computational studies show that effective inhibitors exploit deep hydrophobic enclosure augmented by a limited set of directional polar contacts. The monoterpenoid derivatives proposed in this study reproduce this binding motif. Molecular docking places them in a nonpolar pocket formed by VAL66, LEU68, ALA101, LEU184, LEU186, and aliphatic residues of chain B (LEU515, MET548, LEU554, and LEU558). Additional π-stacking or π-alkyl contacts are predicted with PHE193, PHE194, and TYR517, while ARG64 provides a complementary polar anchor. This interaction pattern aligns closely with the binding modes documented for established entry inhibitors [15,16,17] and supports further evaluation of the new compounds in EBOV pseudotype or live-virus assays.

### 2.4. Molecular Dynamics Simulation Results

**Figure 3:**
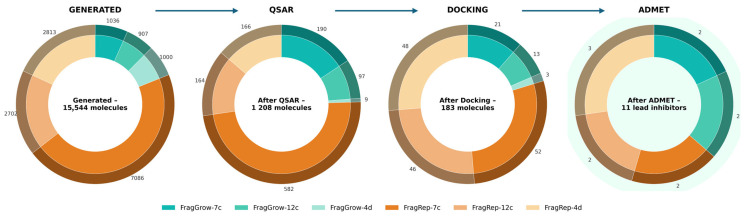
Sequential screening pipeline from generation to final selection.

After QSAR, molecular docking, and ADMET filtrations, eleven molecules were selected as the most potent and safest. Figure 3 provides a schematic overview of the multistep filtration pipeline and summarizes the corresponding outcomes at each stage.

**Figure 4:**
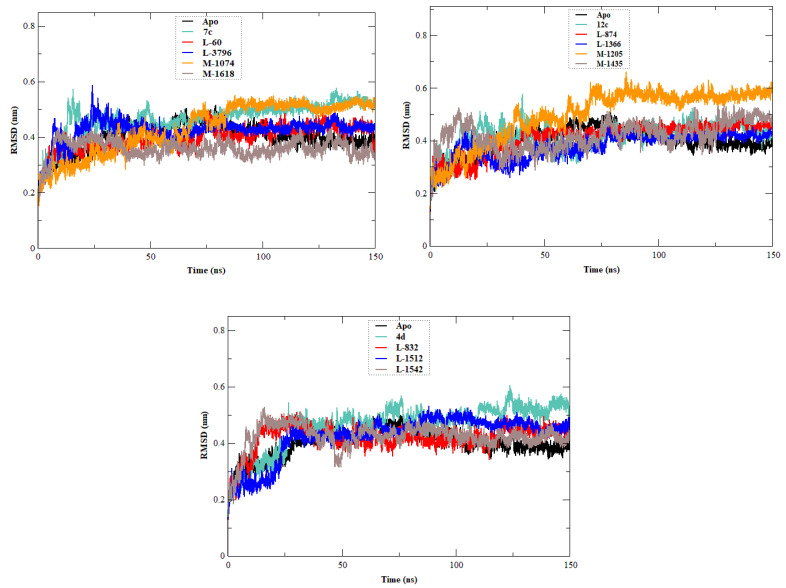
Time-dependent RMSD of c-α backbone of the EBOV-GP-apo, leads (**7c**, **12c**, and **4d**), and designed compounds. The figure is composed of three graphs, each corresponding to one lead compound (**7c**, **12c**, or **4d**) and its derived designed analogs, together with the apo system, to compare the structural stability of the different protein–ligand complexes.

**Figure 5:**
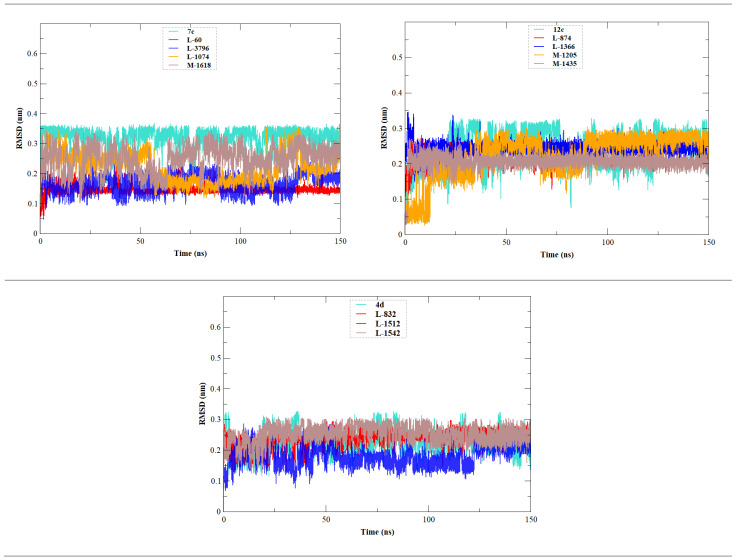
RMSD profiles of ligand heavy atoms during the 150 ns molecular dynamics simulations. The figure is divided into three panels corresponding to the lead compounds **7c**, **12c**, and **4d** and their respective designed analogs generated from each scaffold.

**Figure 6:**
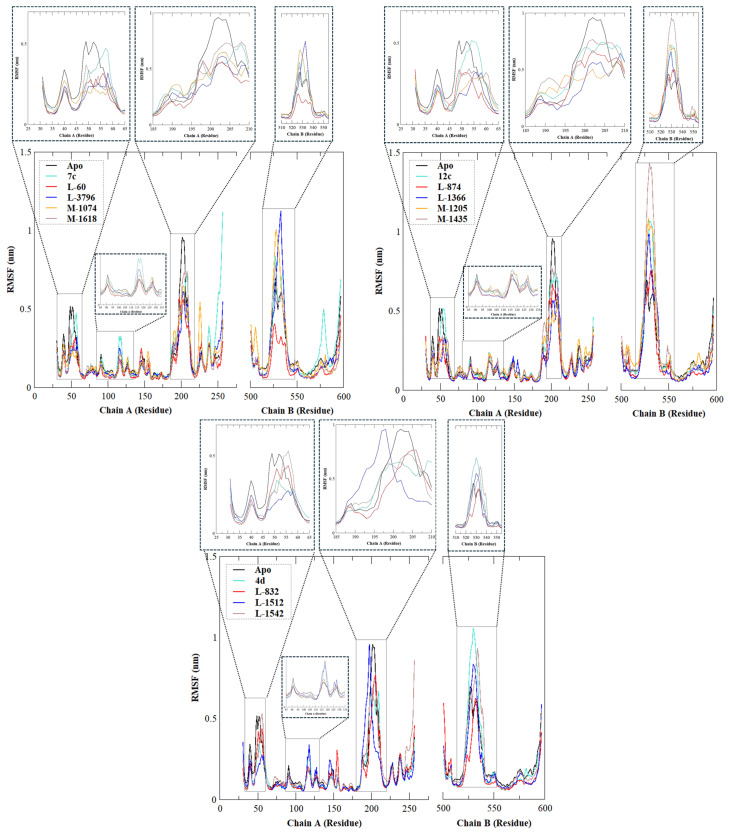
RMSF of c-α atoms of EBOV-GP -apo, leads (**7c**, **12c**, and **4d**), and designed compounds. The figure is divided into three graphs, each representing one lead compound (**7c**, **12c**, or **4d**) together with its derived designed analogs and the apo protein, highlighting variations in local flexibility of the protein residues upon ligand binding.

**Figure 7:**
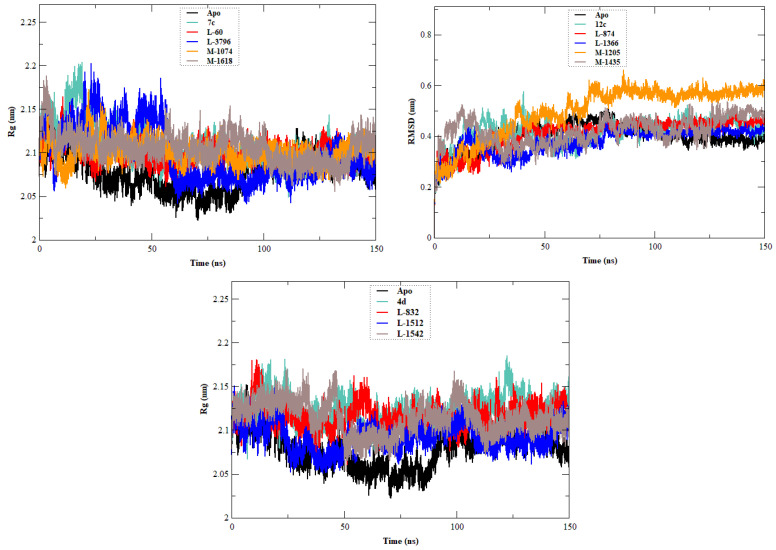
Plot of Rg vs. time for the EBOV-GP-apo, leads (**7c**, **12c**, and **4d**), and designed compounds. The figure is composed of three panels, each presenting the system associated with one lead compound (**7c**, **12c**, or **4d**) together with its corresponding designed analogs and the apo form of the protein, allowing comparison of the global compactness of the different protein systems during the simulation.

**Figure 8:**
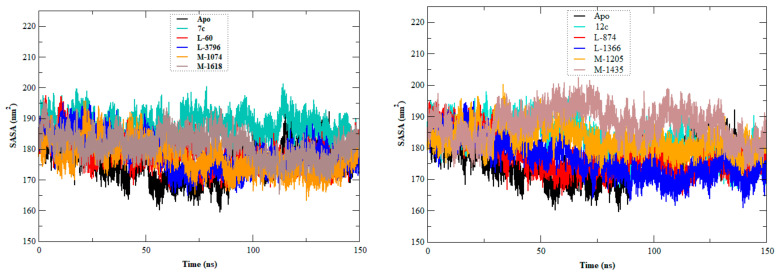

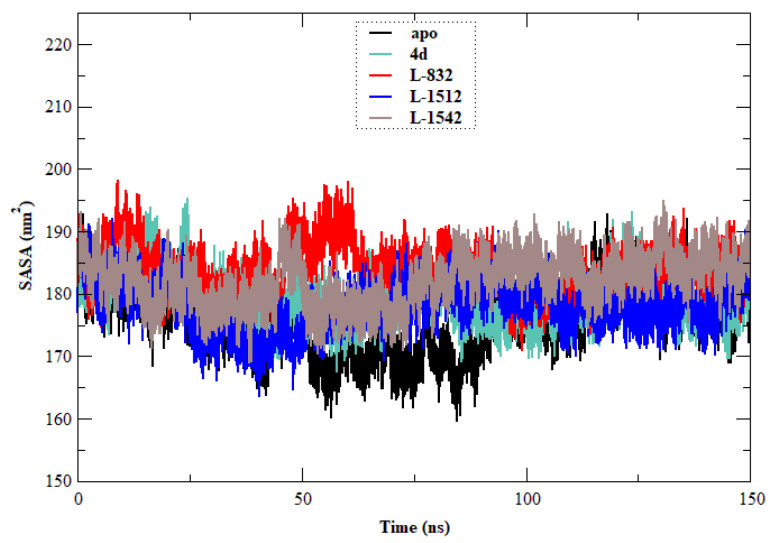
SASA plots of the EBOV-GP-apo, leads (**7c**, **12c**, and **4d**), and designed compounds. The figure consists of three panels, each corresponding to one lead compound (**7c**, **12c**, or **4d**) along with its derived designed analogs and the apo protein, allowing comparison of changes in protein surface exposure throughout the simulation.

The stability, flexibility, and dynamic behavior of the qualified inhibitors were further assessed with comprehensive MD simulations. Five key metrics were examined: RMSD, RMSF, Rg, SASA, and HB. A summary of the average values of these structural metrics is presented in Table 4, while Figure 4, Figure 5, Figure 6, Figure 7 and Figure 8 illustrate their evolution over time.

RMSD is one of the most metrics that outlines the backbone residues stability over time (Figure 4). The apo EBOV-GP structure had an RMSD value of approximately 0.400 ± 0.052 nm. As expected, the reference compounds **7c**, **12c**, and **4d** were more invasive, showing a backbone RMSD of 0.467 ± 0.063 nm, 0.414 ± 0.054 nm, and 0.459 ± 0.082 nm, respectively.

Among the newly designed compounds, **M-1618** showed notable improvement in stability with the lowest RMSD (0.358 ± 0.035 nm), followed by **L-1366** (0.376 ± 0.511 nm) and **L-60** (0.396 ± 0.047 nm), all of which outperformed the apo structure. In addition, a broader set of designed molecules displayed RMSD values lower than those of the lead compounds. These included **L-3796**, **M-1074**, **L-832**, **L-1512**, **L-1542**, **M-1435**, and **L-874**, all of which showed reduced deviations compared to at least one of the reference complexes (**7c**, **12c**, or **4d**). Collectively, these results indicate that many of the newly designed compounds induce less backbone fluctuation than the original lead compounds, supporting their improved stabilizing effect on EBOV-GP.

To further evaluate the stability of the ligands within the EBOV-GP binding pocket, the RMSD of the ligand was assessed throughout 150 ns simulation (Figure 5). Analysis of these trajectories provides insight into the conformational behavior and positional stability of the ligands within the active site during the MD simulation period. The RMSD profile indicates that the compound **L-60** displays the most stable behavior, with minimal fluctuations over the entire trajectory, indicating a well-maintained binding orientation within the pocket. Similarly, **L-3796**, **M-1435**, and **L-1512** also exhibited relatively stable RMSD profiles with limited deviations, indicating consistent ligand arrangement within the binding site. In contrast, **7c**, **12c**, **4d**, **L-1542**, **M-1618**, **L-874**, and **L-1366** presented higher RMSD variations, reflecting greater conformational mobility inside the binding pocket before gradually stabilizing toward the final stages of the simulation.

RMSF enables us to estimate the residue-wise movement concerning precisely the Cα atoms. The apo protein provided an average RMSF value of 0.186 ± 0.167 nm, whereas the reference compounds **7c** (0.202 ± 0.184 nm), **12c** (0.189 ± 0.191 nm), and **4d** (0.174 ± 0.182 nm) increased the flexibility of some regions, especially in loop portions (Figure 6). Notably, **L-60** exhibited the lowest RMSF value (0.137 ± 0.107 nm), indicating enhanced structural stability and reduced local flexibility throughout the simulation. This behavior is reflected by its RMSF profile remaining consistently below those of the other complexes, particularly within the loop regions proximal to the binding pocket, which are typically associated with higher mobility (residues 35–65, 190–210, and 515–555). Similarly, **L-3796**, **M-1074**, **M-1618**, **L-874**, **L-1366**, **M-1205**, **L-832** and **L-1512** also displayed lower RMSF values compared with the apo protein and the reference lead complexes, suggesting that ligand binding contributes to stabilizing the binding-site residues and reducing their conformational fluctuations. These results indicate that these compounds promote a more rigid and stable protein environment around the active site.

These observations suggest that the designed ligands enhance the stability of the conformation at the interface of binding.

To evaluate the tightness of EBOV-GP, Rg calculations were performed (Figure 7). The apo form exhibited an average Rg value of 2.075 ± 0.091 nm. Each of the three leads contributed to increasing the Rg values, with **7c** measuring 2.103 nm, **12c** 2.104 nm, and **4d** 2.119 nm. This indicates there was some degree of protein structure expansion upon ligand binding. The designed analogs, however, generally maintained or reduced this effect. Remarkably, **L-1366** and **L-874** (both 2.082 nm) preserved protein compactness more effectively. Additionally, several compounds (**L-60**, **L-3796**, **M-1074**, **M-1618**, and **L-1512**) exhibited lower Rg values than those observed for the lead compounds. Such results consolidate the assumption that the newly developed compounds oscillate the structural perturbation on the target protein.

SASA quantitates the solvent exposure of the protein surface, which reflects the ligand-induced conformational change (Figure 8). The SASA of the apo form was 175.591 ± 8.876 nm^2^, and greater solvent exposure was evident for **7c**, **12c**, and **4d** (187.362, 182.042, and 179.262 nm^2^, respectively). The newly designed compounds **L-60**, **L-3796**, **M-1074**, **L-874**, **L-1366**, and **L-1512** exhibited SASA values lower than the lowest value observed among the lead compounds. These results show that these new molecules are causing more compact protein structures with less surface exposure to solvent molecules.

**Figure 9:**
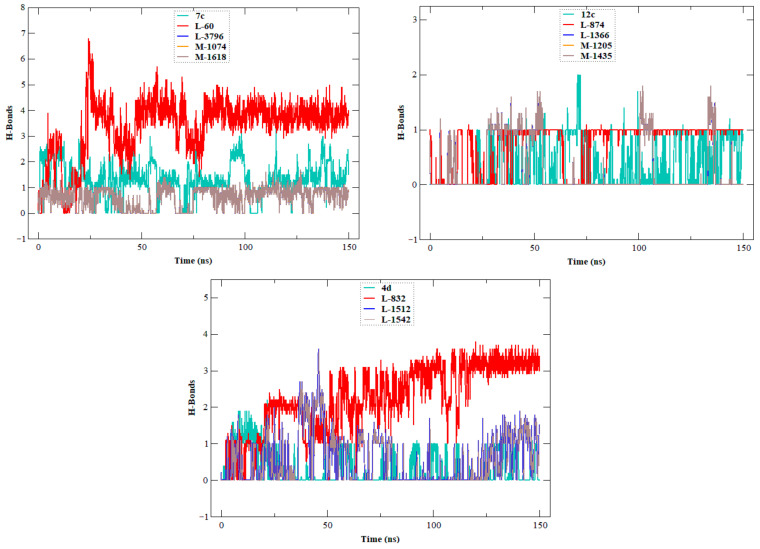
HB plots of the EBOV-GP leads (**7c**, **12c**, and **4d**), and EBOV-GP designed compound systems. The figure contains three panels, corresponding to the systems with the lead compounds (**7c**, **12c**, and **4d**) and their respective designed analogs, illustrating the formation and stability of hydrogen bonds throughout the simulation.

To gain a better understanding of the binding behavior of the newly designed compounds in comparison to their respective reference compounds (**7c**, **12c**, and **4d**), hydrogen bonds were analyzed during the 150 ns (Figure 9). The compounds **L-60** and **L-832** showed a strong binding behavior, forming an average of 5–8 hydrogen bonds.

### 2.5. MM-PBSA Binding Free Energy

The MM-PBSA method was employed to estimate the binding free energies of the selected compounds toward EBOV-GP by decomposing the interaction into molecular mechanics (van der Waals and electrostatic) and solvation (polar PB and nonpolar surface) contributions. More negative ΔH_total_ values indicate stronger predicted ligand–protein association. The reported values represent averages over multiple MD trajectory snapshots and are summarized in Table 5. Among the evaluated systems, **L-60** exhibited the most favorable binding free energy (ΔH_total_ = −33.92 ± 0.17), followed by **L-832** (−29.33 ± 0.18), both outperforming the corresponding lead complexes (**7c**, **12c**, and **4d**). For these ligands, favorable binding is primarily driven by a strong van der Waals contribution together with a favorable gas-phase electrostatic term that is partly offset by the polar solvation penalty, consistent with typical MM-PBSA behavior. A second tier of compounds showed binding free energies comparable to the lead compounds, including **7c** (−22.65 ± 0.20), **M-1205** (−22.52 ± 0.19), **12c** (−22.34 ± 0.22), and **L-1542** (−22.20 ± 0.13), whereas **L-1366** (−4.69 ± 0.24) displayed a markedly less favorable energetic profile. Overall, the MM-PBSA analysis prioritizes **L-60** and **L-832** as the most promising candidates for follow-up optimization.

### 2.6. DFT Study

#### 2.6.1. Geometry Optimization

**Figure 10:**
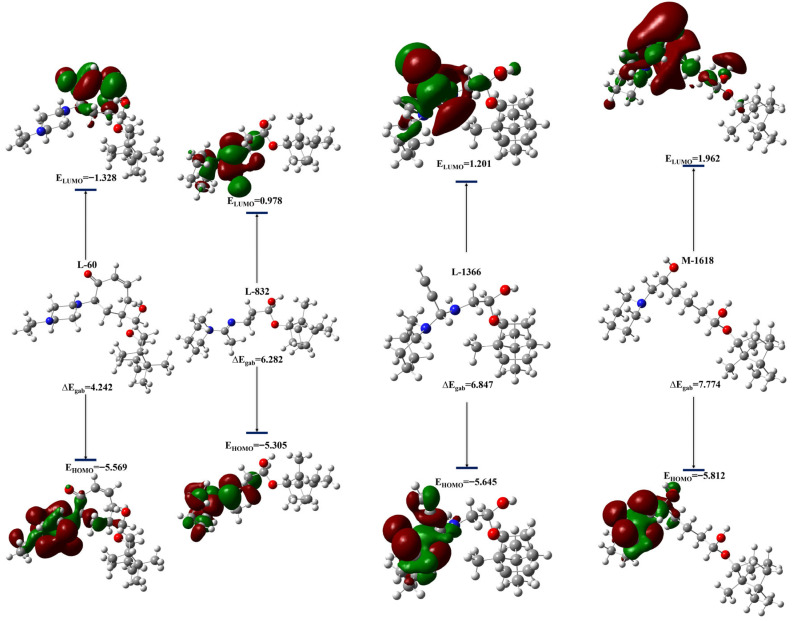
Optimized structures and frontier molecular orbitals (HOMO-LUMO) of the top four compounds **L-60**, **M-1618**, **L-1366**, and **L-832**, at the B3LYP/6-31G(d,p) level in the gas phase.

A quantum study of the four main most representative compounds from MD simulations and MM-PBSA results was carried out using the DFT method at the B3LYP/6-31G(d,p) level in the gas phase, in order to deepen the understanding of their electronic stability. Figure 10 and Figure S4 show the optimized molecular structures of the compounds, superimposed onto the distribution of the electron density of the HOMO and LUMO frontier orbitals, thus revealing their location in each molecule.

#### 2.6.2. Analysis of Frontier Molecular Orbitals and Global Reactivity Descriptors

FMO analysis offers a rigorous metric for assessing ligand reactivity by examining the energies of the highest occupied (HOMO) and lowest unoccupied (LUMO) molecular orbitals. A high-energy HOMO denotes pronounced electron-donor capacity, whereas a low-energy LUMO reflects an enhanced propensity for electron uptake. The HOMO-LUMO gap (ΔEg) therefore serves as a proxy for chemical lability: a reduced gap usually correlates with high reactivity, better binding affinity, and diminished intrinsic stability, while a wider gap implies the opposite. Conceptual-DFT descriptors (Table 6) reveal that the newly designed analogs **L-832**, **L-1366**, and **M-1618** present the electronic balance of the original leads **4d**, **7c**, and **12c** (ΔE around 6.0 and 6.2 eV; hardness η around 3.0 eV; electrophilicity index ω around 1.10). This similarity indicates that these compounds preserve the electronic balance of the lead structures while introducing favorable structural and dynamic features observed in the MD simulations. This profile indicates adequate reactivity, making it possible to interact effectively with the key residues of the active EBOV-GP pocket while preserving good electronic stability. The mode of action is most likely based on strong and directional noncovalent interactions, in particular multiple hydrogen bonds and ionic interactions with the charged residues. Favorably, the compound **L-60** displays a markedly contracted energy gap (4.242 eV), minimal hardness, pronounced softness, and a high electrophilicity index (ω = 2.804) consistent with an elevated polarizable electron-accepting scaffold. Lower chemical hardness and high softness often correlate with stronger binding affinities and lower HOMO-LUMO gaps, indicating that chemical softness is a key driver for enhanced binding interactions. These features illustrate higher electronic polarizability and an enhanced ability to participate in intermolecular interactions which may facilitate charge-transfer processes and strengthen electrostatic and hydrogen-bonding interactions within the active site. However, it is important to emphasize that the HOMO-LUMO gap is not used as a standalone predictor of binding affinity, but rather as a complementary descriptor that supports the observed trends from MM-PBSA and molecular dynamics simulations. Such an electronic signature distinguishes **L-60** from the other candidates and aligns well with its superior MM-PBSA binding free energy and stable dynamic behavior. Although no strict quantitative correlation is expected due to the different theoretical frameworks involved between quantum mechanical descriptors and classical free energy calculations, these results highlight a consistent trend linking electronic properties to binding performance.

Frontier molecular orbital and conceptual-DFT parameters show that the newly prioritized ligands match the electronic requirements for productive binding to EBOV-GP. **L-832** showed the highest HOMO energy and the greatest predicted electron-transfer fraction (ΔN = 0.423), classifying it as the strongest electron donor in the set, an attribute that should reinforce electrostatic or hydrogen-bond contacts with electron-poor residues in the pocket. This donor-dominated behavior complements the acceptor-driven electronic profile observed for **L-60**. **L-1366** and **M-1618** display wider HOMO-LUMO gaps and higher hardness, implying greater intrinsic stability and slightly lower polarizability than the parent leads. Such characteristics may contribute to improved selectivity and controlled reactivity upon binding. All four molecules, however, maintain moderate softness, negative chemical potentials, and positive ΔN values, ensuring efficient charge redistribution once bound. These electronic results explain the biological activity observed by a synergy between stability and reactivity. The moderate softness and the negative chemical potentials confer an intrinsic robustness, avoiding rapid degradation in vivo, while the positive ΔN values facilitate an efficient charge transfer towards the target in the biological medium. This balance promotes both powerful and selective inhibition, while the original leads remain useful electronic benchmarks.

#### 2.6.3. Molecular Electrostatic Potential Analysis

**Figure 11:**
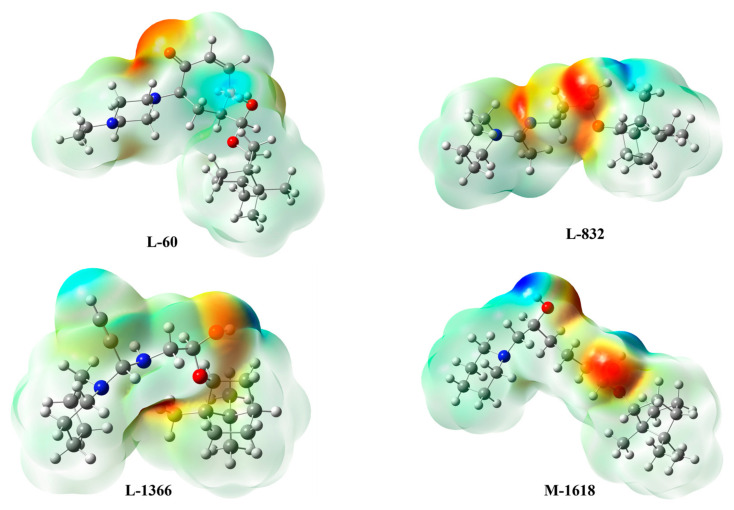
MEP surface analysis of the top four compounds **L-60**, **L-832**, **L-1366**, and **M-1618**. The color scale from red (electron-rich regions) to blue (electron-poor regions) illustrates the charge distribution and potential interaction sites.

MEP maps (Figure 11 and Figure S5) confirm the suitability of the ligands for EBOV-GP targeting. Nitrogen atoms in **L-832**, **L-1366**, and **M-1618** generate continuous regions of negative potential, marking strong nucleophilic sites capable of salt-bridge or hydrogen-bond formation with cationic or electrophilic residues. Oxygen atoms add further negative regions, reinforcing their role as hydrogen-bond acceptors. The near-identical MEP contours of these compounds suggest comparable binding orientations and predictable structure–activity relationships. **L-60** exhibits a similar pattern, with pronounced negative potential around its nitrogen centers and compensating positive lobes near electronegative substituents, consistent with its high affinity. The lowest electrostatic potential, of an electropositive nature, is localized around the hydrogen atoms linked to the N and O heteroatoms. The strong polarization of the N-H and O-H bonds gives them a marked positive partial charge, designating them as hydrogen bond donor sites capable of interacting with electronegative acceptors of the biological medium. The combined analysis of global reactivity descriptors and MEP surfaces provides a consistent and coherent interpretation. The new ligands pair adequate electronic softness with strategically positioned nucleophilic domains, positioning them as credible successors to the first-generation leads for further optimization as EBOV-GP entry inhibitors. Overall, the DFT analysis provides electronic-level validation of the stability and binding trends observed in the MD and MM-PBSA studies, reinforcing **L-60** and **L-832** as leading candidates while supporting **L-1366** and **M-1618** as structurally robust scaffolds for future refinement.

## 3. Materials and Methods

### 3.1. Dataset Curation and Descriptor Preprocessing

A dataset of 55 terpenoid derivatives with experimental activities against EBOV-GP was retrieved from a recently published article [18]. 2D structures were first sketched in ChemDraw (v23.1.2.7) [19] and then energy-minimized using the MMFF94 force field within ChemDraw 3D. This provides an energetically favorable input for molecular descriptor calculations. The molecular structures together with their IC_50_ and converted pIC_50_ values are given in Table S1. Over 5666 different molecular descriptors covering 2D, 3D, topological, geometrical, and physicochemical classes were computed via AlvaDesc software v3.0.0 [20]. This extensive pool was pretreated to exclude inter-correlated descriptors with a correlation coefficient of 0.95, constant or near-constant ones, and eliminate the null or missing values.

### 3.2. QSAR Model Construction and Validation

Based on pIC_50_ distributions, the monoterpenoid dataset was split into a training set (80%) and an external validation set (20%). In model building, a Genetic Algorithm-based Multiple Linear Regression (GA-MLR) was applied using QSARINS software v2.2.4 [21]. The chosen algorithm optimizes both the selection of variables and the parameters of the model through evolutionary selection over several generations. Model validation was assessed according to the Organization for Economic Co-operation and Development (OECD) principles, including statistical criteria such as R^2^_train_, Q^2^_LOO_, R^2^_test_, Q^2^_F1–F3_, and the concordance correlation coefficient (CCC). To ensure robustness, Y-randomization tests (1000 iterations) were applied to verify that the observed model performance did not arise from chance correlations. A Williams plot was used to define the applicability domain (AD), identifying potential structural outliers beyond the threshold leverage value (h*) [22,23,24]. Our earlier studies provided a detailed account of these processes and criteria used to validate the QSAR models [25,26,27].

### 3.3. Structure Preparation and Molecular Docking

A molecular docking study was carried out to explore the binding interactions between the designed analogs and EBOV-GP. The EBOV-GP structure with PDB ID 7M2D was retrieved from the Protein Data Bank, chosen due to the availability of high-resolution crystallographic data (2.70 Å) and the presence of a co-crystallized ligand. This structure was incomplete and required homology-based loop modeling to reconstruct two missed regions (ALA189-SER211 and ILE281-GLU287). SWISS-MODEL and ProMod3 were used for homology reconstruction, with QMEAN and GMQE scores guiding the model selection as described in our prior published work [25]. Protein and ligand files were then prepared using AutoDockTools v1.5.7 [28]. Protein preparation involved the removal of water molecules, co-factors, and the native ligand, followed by the addition of polar hydrogens and Kollman charges. For the ligands, polar hydrogens were added, and torsional flexibility was assigned to all rotatable bonds. Both the protein and ligand structures were converted into PDBQT format for docking. The docking grid box was centered on the co-crystallized ligand (YPS) at x = −44.98, y = 14.38, and z = −7.31, with dimensions of 30 Å^3^. Docking was performed in AutoDock Vina software v1.1.2 [29], applying an exhaustiveness of 32 to enhance the accuracy of conformational sampling. The resulting binding poses and the key molecular interactions within the EBOV-GP active site were analyzed and visualized using Discovery Studio Visualizer (v25.1.0.24284), and PyMOL software (v3.1.5.1, edu, 2025) [30,31].

### 3.4. Fragment-Based Design and Library Generation

Three terpenoid scaffolds, **7c**, **12c**, and **4d**, served as the starting points for analog generation. Scaffold diversification used the structure-guided fragment-replacement engine FragRep [32]. The program breaks each ligand at rotatable bonds and swaps user-defined regions with fragments drawn from a library of over 700,000 structures, keeping the ligand’s core pose in the EBOV-GP pocket. Candidate analogs were scored against the protein and screened for synthetic tractability, Lipinski compliance, and structural soundness, to select the best ones.

Complementary fragment growth was carried out with FragGrow server [33]. From designated growth vectors, fragments were added stepwise and assessed for steric fit, hydrogen-bonding capacity, and hydrophobic occupancy within the binding site. Each new molecule was energy-minimized and ranked by a composite shape/binding score. Molecules with optimal predicted interactions and conformational fit were retained and advanced to subsequent QSAR evaluation and docking workflows. This process yielded 15,544 unique compounds (Files S7–S12), which were subsequently transformed into 3D formats and minimized with the MMFF94 force field using RDKit 2025.09.6 [34]. The MMFF94 force field was selected due to its reliable parametrization for small organic molecules and its proven performance in reproducing accurate geometries of drug-like compounds. In addition, it offers an efficient balance between computational cost and accuracy, making it suitable for ligand preparation prior to QSAR modeling and molecular docking. The generated compounds constituted the virtual library for predictive evaluation.

### 3.5. ADME Prediction and Toxicity Profiling

ADMET predictive assessment serves to ensure that only pharmacologically safe and synthetically accessible compounds were prioritized for molecular dynamics and energetic evaluations. In this context, pharmacokinetic and toxicity parameters were evaluated for the filtered compounds using the pkCSM web server, which applies graph-based approaches to predict a wide range of ADMET endpoints [35]. For absorption properties, human intestinal absorption (HIA), Caco-2 cell permeability, and aqueous solubility (logS) were calculated. Distribution parameters included blood–brain barrier (BBB) penetration. Metabolism was assessed by identifying potential cytochrome P450 enzyme inhibitors, specifically focusing on some main isoforms (CYP2C19 and CYP2C9), to anticipate any risk of metabolic interactions. Excretion properties were predicted through clearance. For the toxicological endpoints, the predictions included AMES mutagenicity, hepatotoxicity, genotoxicity and carcinogenicity. Additionally, the synthetic accessibility of each compound was predicted using the ADMETlab 2.0 web server [36], providing scores on a scale from 1 (easy to synthesize) to 10 (very difficult). Only compounds that passed all filters for drug-likeness demonstrated high intestinal absorption, acceptable solubility, no major metabolic red flags, and non-toxic profiles were selected for further molecular dynamics simulations.

### 3.6. Molecular Dynamics Simulations

To study the structural stability of the selected ligand-bound EBOV-GP complexes, all-atom molecular dynamics simulations were performed using GROMACS 2021.3 [37]. System preparation, such as topology file generation and input parameter creation, was accomplished using the CHARMM-GUI web server [38] with the CHARMM36 force field [39]. Each protein–ligand system was inserted into a rectangular periodic box and solvated using the TIP3P water model [40]. Sodium and chloride ions were added using a Monte Carlo procedure to neutralize the system and to achieve a physiological ionic strength of 0.15 M. The Verlet cutoff technique was applied for non-bonded interactions, and a 12 Å cutoff was applied to both van der Waals and short-range electrostatic interactions. Long-range electrostatics were computed using the Particle Mesh Ewald (PME) technique [41]. All bonds involving hydrogen atoms were constrained by the LINCS algorithm for stable integration [42]. Energy minimization was conducted using the steepest descent algorithm for up to 50,000 steps or until the system reached a convergence criterion of 10.0 kJ/mol/nm. The minimized structures were then equilibrated in two phases: an initial 500 ps NVT simulation at 303 K regulated by the Nose–Hoover thermostat and a 500 ps NPT simulation at 1.01325 bar using the Parrinello–Rahman barostat [43,44]. The production phase was runed for 150 ns, during which the conformational behavior and dynamic stability of the complexes were tracked. The calculated parameters included root mean square deviation (RMSD), root mean square fluctuation (RMSF), radius of gyration (Rg), and solvent accessible surface area (SASA), which were used to evaluate structural stability, flexibility, compactness, and exposure to solvent environment, respectively. In addition, the number and persistence of hydrogen bonds formed between the ligands and the EBOV-GP binding site were quantified to assess interaction stability. All analyses were performed using the GROMACS version 2021.3 built-in utilities, and the data was plotted using the Xmgracesoftware version 5.1.22 [45].

### 3.7. MM-PBSA Binding Free Energy Calculations

The binding free energy was calculated using the MM-PBSA approach. This method offers the combination of molecular mechanics and continuum solvent models to calculate the binding free energy. The binding free energy (ΔG_binding_) is computed according to the following equation:

ΔG_binding_ = ΔG_PL_ − [ΔG_P_ + ΔG_L_] (2)

where PL, P and L represent the free energy of state of protein–ligand, protein and ligand, respectively, and are estimated by using the following expression:

ΔG_binding_ = ΔE_gas_ + ΔG_solv_ = ΔE_vdw_ + ΔE_ele_ + ΔG_polar_ + ΔG_nonpolar_ (3)

In this equation, ΔE_gas_ represents the gas-phase potential energy arising from van der Waals and electrostatic interactions, whereas ΔG_solv_ accounts for the solvation free energy, including polar and nonpolar contributions. The MM-PBSA calculations were performed using gmx_MMPBSA v1.6.4 2024 [46]. These calculations were performed over the last 100 ns of the trajectory. A total of 10,000 snapshots were uniformly extracted from 150,000 frames using an interval of 10. The gmx_MMPBSA_ana tool (v1.4.3, 26 May 2021) was used to display the outcomes of the gmx_MMPBSA calculations.

### 3.8. Computational Investigations and MEP Analysis

In this study, quantum chemical calculations were performed using the Gaussian 09 program [47]. The hybrid density functional B3LYP [48] was employed with the 6-31G(d,p) basis set for all atoms, with geometries optimized in the gas phase. Complete geometric optimizations have been carried out in order to identify the most stable conformations. For each structure, an analysis of the vibrational frequencies was then carried out to confirm that the optimized geometries correspond to true minima on the potential energy surface, which is evidenced by the absence of imaginary frequencies. Gaussian 09 default convergence criteria were applied during the computations process. Main electronical characteristics such as frontier molecular orbitals (FMOs), global reactivity indexes, and Molecular Electrostatic Potential (MEP) were examined on the optimized structure to gain deeper insights on the molecules’ stability.

## 4. Conclusions

The present study establishes an integrated CADD workflow for the discovery of small-molecule inhibitors targeting the Ebola virus surface glycoprotein (EBOV-GP). The pipeline commences with the construction of a rigorously validated QSAR model, which serves as a high-throughput filter for a large virtual library generated by fragment-growing and fragment-replacement algorithms. Molecules achieving favorable QSAR scores were subsequently evaluated by molecular docking against a homology-derived EBOV-GP structure, and only those exhibiting strong binding energies and key intermolecular contacts are retained for further analysis.

The qualified candidates undergo ADMET assessment. All shortlisted compounds display desirable pharmacokinetic attributes, most notably high predicted gastrointestinal absorption, minimal toxicity liabilities, and acceptable synthetic accessibility, thereby meeting essential criteria for oral drug development. Eleven top-ranked molecules are then subjected to explicit-solvent MD simulations. Throughout the trajectories each compound maintains a stable, low-energy pose within the EBOV-GP binding pocket and exhibits limited conformational drift, confirming both structural integrity and persistent intermolecular interactions over time.

Among the designed molecules, from the three principal scaffolds (**7c**, **12c**, and **4d**), the four analogs **M-1618**, **L-1366**, **L-832**, and **L-60** emerged as the most promising candidates based on their predicted biological activity and favorable interaction patterns within the EBOV-GP binding pocket. These compounds displayed predicted IC_50_ values of 0.0589 µM, 0.0767 µM, 0.0196 µM, and 0.092 µM, respectively, and maintained stable binding conformations during the 150 ns MD simulations, as supported by consistent RMSD, RMSF, and compactness profiles. Furthermore, MM-PBSA calculations confirmed favorable binding free energies, while DFT analyses revealed electronic properties and frontier orbital distributions consistent with enhanced interaction capability with the target protein. Notably, the results highlight the significant potential of terpenoid-based chemical scaffolds in antiviral drug discovery, given their structural diversity, natural origin, and capacity to form stable interactions with viral targets. In particular, **L-60** and **L-832** appear as the most promising EBOV-GP inhibitors, while **L-1366** and **M-1618** represent robust scaffolds for further optimization.

Regarding the synthetic accessibility, the four most promising compounds have SA scores that fall within a tolerable range (4.739–5.384), which demonstrates the moderate synthetic complexity of the drug-like scaffolds. Given that **L-832** has the highest predicted potency (IC_50_ = 0.0196 μM, pIC_50_ = 7.7077), and its SA score of 4.975 suggests no major synthetic penalty compared to the others, it is therefore prioritized for synthesis. **M-1618** is an interesting alternative due to its lowest synthetic accessibility score (4.739) while keeping high predicted potency (IC_50_ = 0.0589 μM, pIC_50_ = 7.2300), facilitating its synthesis. The compounds **L-1366** (SA = 5.324, IC_50_ = 0.0767 μM, pIC_50_ = 7.1152) and **L-60** (SA = 5.384, IC_50_ = 0.092 μM, pIC_50_ = 7.0373) have slightly higher SA scores, though they remain within a reasonable complexity range and display strong dynamic and energetic performance in later simulations. The results show that rationally choosing **L-832** and **M-1618** for an initial experimental set is advisable, followed by **L-1366** and **L-60**, thereby balancing predicted potency with synthetic tractability and computational guidance.

Collectively, these findings underscore the value of a multi-tiered in silico strategy, spanning QSAR modeling, fragment-based design, docking, ADMET profiling, and MD simulations for rapidly prioritizing high-quality antiviral leads. The compounds derived from the **7c** and **12c** scaffolds constitute a compelling starting point for experimental validation and subsequent lead optimization toward next-generation EBOV therapeutics.

## Figures and Tables

**Table 1:** Assessment of the structural quality of the EBOV-GP modeled structure.

Overall Quality Factor	Ramachandran Plot Statistics (%)				Structure Z-Scores	
	Most Favored Regions	Additionally Allowed Regions	Generously Allowed Regions	Disallowed Regions	1st Generation Packing Quality	Ramachandran Plot Appearance
**88.182**	90.2	9.2	0.0	0.6	−1.688	−1.175

**Table 2:** Physiochemical properties of the newly designed compounds.

		FragGrow				FragRep						
		**7c**		**12c**		**7c**		**12c**		**4d**		
	**Cmpd.**	**M-1074**	**M-1618**	**M-1205**	**M-1435**	**L-3796**	**L-60**	**L-1366**	**L-874**	**L-1512**	**L-832**	**L-1542**
Physicochemical and ADME properties	MW	349.559	354.555	315.457	380.326	404.595	495.688	348.531	348.531	337.528	337.528	340.532
	LogP	4.438	2.136	4.425	4.757	3.078	3.284	2.961	2.961	2.447	1.911	1.436
	HBA	3	3	3	3	5	5	4	4	3	2	4
	HBD	1	3	1	1	1	2	2	2	2	2	3
	LogS	−3.845	−3.008	−4.6	−5.302	−3.437	−3.109	−2.446	−2.446	−2.786	−3.095	−2.447
	Caco-2 Permeability	1.155	1.107	2.076	2.165	1.187	1.036	1.189	1.189	1.191	1.207	1.132
	HIA	92.256	97.353	94.776	93.188	94.534	94.54	91.422	91.422	94.05	95.741	98.75
	BBB	0.06	−0.438	0.016	−0.242	−0.02	−0.14	0.279	0.279	0.219	0.272	0.043
	CYP2C19 inhibitor	No	No	Yes	No	No	Yes	No	No	No	No	No
	CYP2C9 inhibitor	No	No	Yes	Yes	No	No	No	No	No	No	No
	Clearance	1.077	1.351	1.049	−0.034	0.767	1.038	1.061	1.061	1.261	1.34	1.192
Medicinal Chemistry	Synthetic accessibility score	4.798	4.739	4.875	5.099	5.387	5.384	5.324	5.324	5.02	4.975	5.056
	Lipinski rule	Accepted	Accepted	Accepted	Accepted	Accepted	Accepted	Accepted	Accepted	Accepted	Accepted	Accepted
	Golden Triangle	Accepted	Accepted	Accepted	Accepted	Accepted	Accepted	Accepted	Accepted	Accepted	Accepted	Accepted
	PAINS	0	0	0	0	0	0	0	0	0	0	0
Toxicity	Genotoxic–carcinogenicity–mutagenicity	0 alert	0 alert	0 alert	0 alert	0 alert	0 alert	0 alert	0 alert	0 alert	0 alert	1 alert
	AMES toxicity	No	No	No	No	No	No	No	No	No	No	No
	Hepatotoxicity	No	No	No	No	No	No	No	No	No	No	No

**Table 3:** Binding affinities and predicted pIC_50_ values of the top eleven ranked ligands with the three lead compounds and toremifene after molecular docking.

Position	Hit ID	Chemical Structure	pIC_50_	Binding Affinity Kcal/mol
	Toremifene	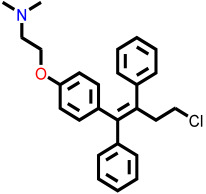		−6.9
	Lead **7c**	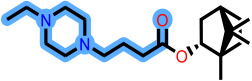	7.0	−7.2
	Lead **12c**	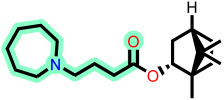	6.7	−7.7
	Lead **4d**	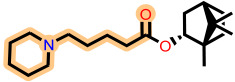	6.7	−7.6
**7c**	**L-60**	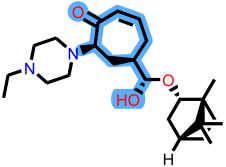	7.0373	−8.6
	**L-3796**	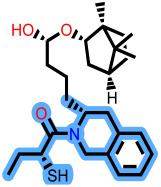	7.0097	−9.0
	**M-1074**	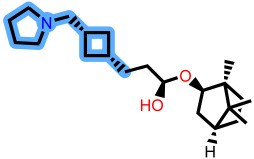	7.5922	−8.2
	**M-1618**	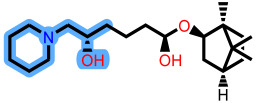	7.2300	−7.7
**12c**	**L-874**	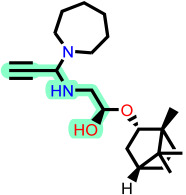	7.1152	−7.9
	**L-1366**	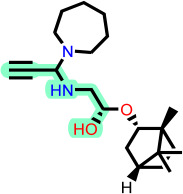	7.1152	−7.9
	**M-1205**	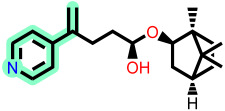	7.1471	−7.9
	**M-1435**	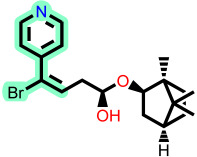	6.5670	−8.1
**4d**	**L-832**	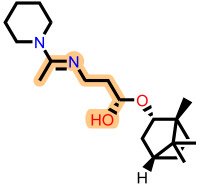	7.7077	−7.6
	**L-1512**	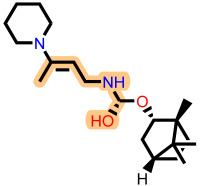	7.7515	−7.6
	**L-1542**	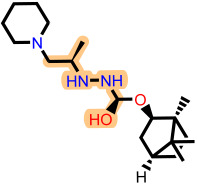	7.0439	−7.7

**Table 4:** Average structural parameters across 150 ns molecular dynamics simulations.

	RMSD (nm)	Ligand RMSD (nm)	RMSF (nm)	Rg (nm)	SASA (nm^2^)
**apo**	0.400 ± 0.052	--	0.186 ± 0.167	2.075 ± 0.091	175.591 ± 8.876
**7c**	0.467 ± 0.063	0.322 ± 0.0278	0.202 ± 0.184	2.103 ± 0.092	187.362 ± 8.346
**L-60**	0.396 ± 0.047	0.146 ± 0.014	0.137 ± 0.107	2.096 ± 0.089	177.950 ± 8.357
**L-3796**	0.421 ± 0.046	0.168 ± 0.029	0.171 ± 0.165	2.095 ± 0.093	178.419 ± 8.956
**M-1074**	0.432 ± 0.093	0.221 ± 0.048	0.185 ± 0.167	2.097 ± 0.089	177.139 ± 8.617
**M-1618**	0.358 ± 0.035	0.250 ± 0.046	0.160 ± 0.155	2.102 ± 0.091	180.800 ± 8.429
**12c**	0.414 ± 0.054	0.241 ± 0.052	0.189 ± 0.191	2.104 ± 0.095	182.042 ± 9.085
**L-874**	0.409 ± 0.059	0.242 ± 0.021	0.155 ± 0.149	2.082 ± 0.092	177.020 ± 8.952
**L-1366**	0.376 ± 0.511	0.242 ± 0.012	0.158 ± 0.157	2.082 ± 0.094	176.207 ± 9.231
**M-1205**	0.495 ± 0.102	0.223 ± 0.062	0.193 ± 0.178	2.117 ± 0.091	182.441 ± 8.339
**M-1435**	0.418 ± 0.056	0.205 ± 0.020	0.207 ± 0.231	2.132 ± 0.094	187.382 ± 8.998
**4d**	0.459 ± 0.082	0.220 ± 0.035	0.174 ± 0.182	2.119 ± 0.091	179.262 ± 8.322
**L-832**	0.416 ± 0.049	0.242 ± 0.023	0.155 ± 0.139	2.111 ± 0.091	183.41 ± 8.349
**L-1512**	0.422 ± 0.163	0.180 ± 0.034	0.163 ± 0.158	2.088 ± 0.089	177.776 ± 8.131
**L-1542**	0.421 ± 0.049	0.251 ± 0.029	0.189 ± 0.178	2.109 ± 0.091	181.816 ± 8.320

**Table 5:** Binding free energy calculated by MM-PBSA for all complexes. All energy values are given in kcal/mol with the average ± standard error of the mean (SEM).

System	ΔE_vdw_	ΔE_ele_	ΔE_pb_	ΔE_surf_	ΔE_gas_	ΔG_solv_	ΔH_total_
**EBOV-GP-7c**	−27.84 ± 0.19	−316.65 ± 2.48	325.69 ± 2.52	−3.85 ± 0.02	−344.49 ± 2.57	321.85 ± 2.5	−22.65 ± 0.2
**EBOV-GP-L-60**	−38.53 ± 0.14	−312.74 ± 1.06	322.07 ± 1.07	−4.73 ± 0	−351.26 ± 1.06	317.34 ± 1.07	−33.92 ± 0.17
**EBOV-GP-L-3796**	−36.83 ± 0.1	−102.57 ± 0.47	122.8 ± 0.4	−4.39 ± 0	−139.4 ± 0.45	118.41 ± 0.4	−20.99 ± 0.14
**EBOV-GP-M-1074**	−26.06 ± 0.2	−433.58 ± 1.63	443.93 ± 1.56	−4.48 ± 0.02	−459.64 ± 1.62	439.45 ± 1.56	−20.19 ± 0.3
**EBOV-GP-M-1618**	−32.76 ± 0.13	−95.75 ± 0.8	110.94 ± 0.83	−3.98 ± 0.01	−128.51 ± 0.83	106.96 ± 0.83	−21.55 ± 0.16
**EBOV-GP-12c**	−37.62 ± 0.22	−74.49 ± 0.86	93.91 ± 0.9	−4.14 ± 0.01	−112.11 ± 0.9	89.77 ± 0.9	−22.34 ± 0.22
**EBOV-GP-L-874**	−32.45 ± 0.12	−247.73 ± 1.57	265.49 ± 1.46	−3.87 ± 0.01	−280.18 ± 1.56	261.62 ± 1.47	−18.56 ± 0.2
**EBOV-GP-L-1366**	−4.43 ± 0.25	−112.16 ± 5.35	112.65 ± 5.3	−0.75 ± 0.04	−116.59 ± 5.42	111.9 ± 5.28	−4.69 ± 0.24
**EBOV-GP-M-1205**	−36.33 ± 0.13	−108.13 ± 0.48	126.3 ± 0.43	−4.37 ± 0.01	−144.45 ± 0.49	121.93 ± 0.43	−22.52 ± 0.19
**EBOV-GP-M-1435**	−37.45 ± 0.1	−14.21 ± 0.21	38.13 ± 0.25	−4.03 ± 0.01	−51.66 ± 0.24	34.1 ± 0.25	−17.56 ± 0.13
**EBOV-GP-4d**	−33.09 ± 0.15	−70.2 ± 0.95	83.43 ± 0.98	−4.12 ± 0.01	−103.28 ± 0.98	79.32 ± 0.98	−23.97 ± 0.16
**EBOV-GP-L-832**	−20.87 ± 0.15	−299.1 ± 1.48	294.08 ± 1.39	−3.45 ± 0.01	−319.96 ± 1.45	290.63 ± 1.38	−29.33 ± 0.18
**EBOV-GP-L-1512**	−32.92 ± 0.18	−174.58 ± 1.49	191.18 ± 1.39	−4.2 ± 0.01	−207.5 ± 1.44	186.98 ± 1.39	−20.52 ± 0.17
**EBOV-GP-L-1542**	−31.98 ± 0.19	−96.32 ± 0.88	109.95 ± 0.99	−3.85 ± 0.01	−128.31 ± 0.99	106.1 ± 0.99	−22.2 ± 0.13

**Table 6:** Global reactivity descriptors of the selected candidates along with the lead compounds at the B3LYP/6-31G(d,p) level in the gas phase.

	L-832	L-1366	M-1618	L-60	Lead 7c	Lead 12c	Lead 4d
**HOMO (ev)**	−5.305	−5.645	−5.812	−5.569	−5.709	−5.590	−5.677
**LUMO (ev)**	0.978	1.201	1.962	−1.328	0.463	0.429	0.481
**Eg**	6.282	6.847	7.774	4.242	6.172	6.020	6.158
**Ionization energy (I)**	5.305	5.645	5.812	5.569	5.709	5.590	5.677
**Electron affinity (EA)**	−0.978	−1.201	−1.962	1.328	−0.463	−0.429	−0.481
**Electronegativity (χ)**	2.163	2.222	1.925	3.449	2.623	2.580	2.598
**Chemical hardness (η)**	3.141	3.423	3.887	2.121	3.086	3.010	3.079
**Chemical potential (μ)**	−2.163	−2.222	−1.925	−3.449	−2.623	−2.580	−2.598
**Softness (σ)**	0.318	0.292	0.257	0.472	0.324	0.332	0.325
**Global electrophilicity (ω)**	0.745	0.721	0.476	2.804	1.115	1.106	1.096
**Electron back-donation (ΔE)**	−0.785	−0.856	−0.972	−0.530	−0.772	−0.752	−0.770
**Fraction of electron transfer (ΔN)**	0.423	0.379	0.372	0.323	0.356	0.372	0.361

## Data Availability

The original contributions presented in this study are included in the article/Supplementary Materials. Further inquiries can be directed to the corresponding authors.

## Supplementary Materials

The following supporting information can be downloaded at: https://www.mdpi.com/article/10.3390/ijms27072987/s1.

## Abbreviations

The following abbreviations are used in this manuscript:

EBOV	Ebola virus
EBOV-GP	Ebola virus glycoprotein
EVD	Ebola virus disease
GP	Glycoprotein
QSAR	Quantitative structure–activity relationship
FBDD	Fragment-based drug design
NPC1	Niemann–Pick C1
CADD	Computer-aided drug design
MD	Molecular dynamics
MM/PBSA	Molecular mechanics/Poisson–Boltzmann surface area
ADMET	Absorption, distribution, metabolism, excretion, and toxicity
RMSD	Root mean square deviation
Rg	Radius of gyration
RMSF	Root mean square fluctuation
SASA	Solvent-accessible surface area
HB	Hydrogen bonds
TOR	Toremifene
YPS	7M2D co-crystalized ligand
DFT	Density functional theory
HOMO	Highest occupied molecular orbital
LUMO	Lowest unoccupied molecular orbital
MEP	Molecular electrostatic potential
