# Supplementary material for: QSAR-Guided and Fragment-Based Drug Design of Monoterpenoid Inhibitors Targeting Ebola Virus Glycoprotein
Source: Int J Mol Sci. 2026 Mar 25;27(7):2987. doi: 10.3390/ijms27072987 (PMC13073486; doi:10.3390/ijms27072987)
Supplement: Supplementary file 1 [file ijms-27-02987-s001.zip › Supplementary Materials.pdf]

Supporting Information for

QSAR Guided and Fragment Based Drug Design of Monoterpenoid inhibitors Targeting Ebola Virus Glycoprotein

Nouhaila Ait Lahcen [1,*], Wissal Liman [2], Saad Zekri [1], Adnane Ait Lahcen [1], Ashwag S. Alanazi [3], Mohammed M. Alanazi [4], Christelle Delaite [5], Mohamed Maatallah [1,*] and Driss Cherqaoui [1,6]

1 Molecular Chemistry Laboratory, Faculty of Sciences Semlalia, Cadi Ayyad University, UCA, Marrakech 40000, Morocco; s.zekri.ced@uca.ac.ma (S.Z.); adnane.aitlahcen.ced@uca.ac.ma (A.A.L.); cherqaoui@uca.ma (D.C.)
2 Bioinformatics Laboratory, College of Computing, University Mohammed VI Polytechnic, Benguerir 43150, Morocco; wissal.liman@um6p.ma
3 Department of Pharmaceutical Sciences, College of Pharmacy, Princess Nourah bint Abdulrahman University, Riyadh 11671, Saudi Arabia; asalanzi@pnu.edu.sa
4 Department of Pharmaceutical Chemistry, College of Pharmacy, King Saud University, Riyadh 11451, Saudi Arabia; mmalanazi@ksu.edu.sa
5 Laboratoire de Photochimie et d'Ingénierie Macromoléculaires (LPIM), Ecole Nationale Supérieure de Chimie de Mulhouse, Université de Haute-Alsace, 68100 Mulhouse, France; christelle.delaite@uha.fr
6 Sustainable Materials Research Center (SUSMAT-RC), University of Mohammed VI Polytechnic, Benguerir 43150, Morocco
* Correspondence: nouhaila.aitlahcen@ced.uca.ma (N.A.L.); m.maatallah@uca.ma (M.M.)

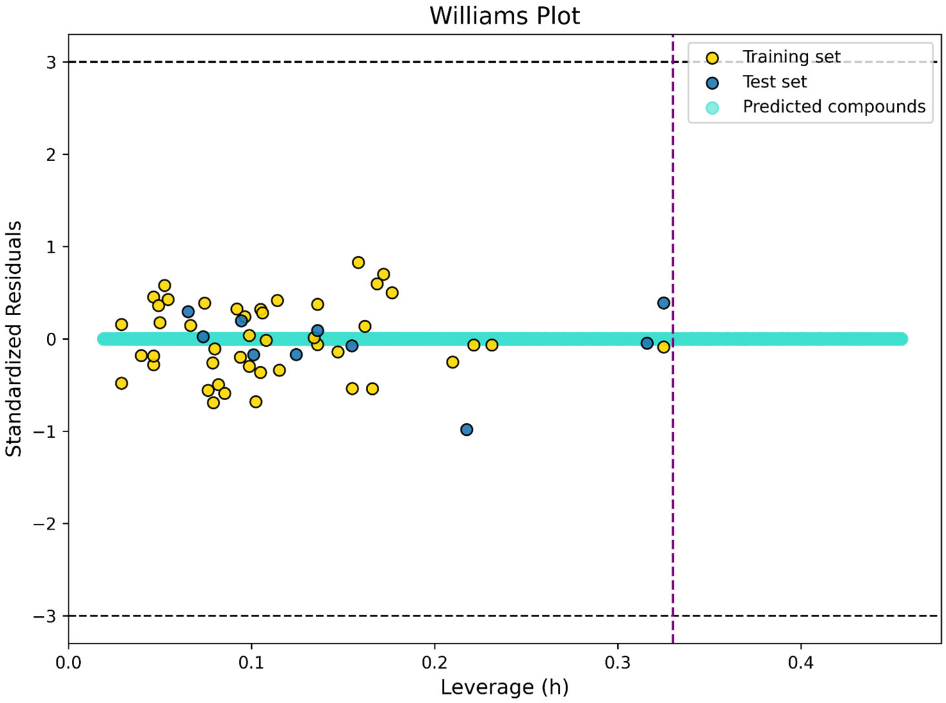

Figure S1. William's plot showing the AD of the newly generated compounds screened by the QSAR model.

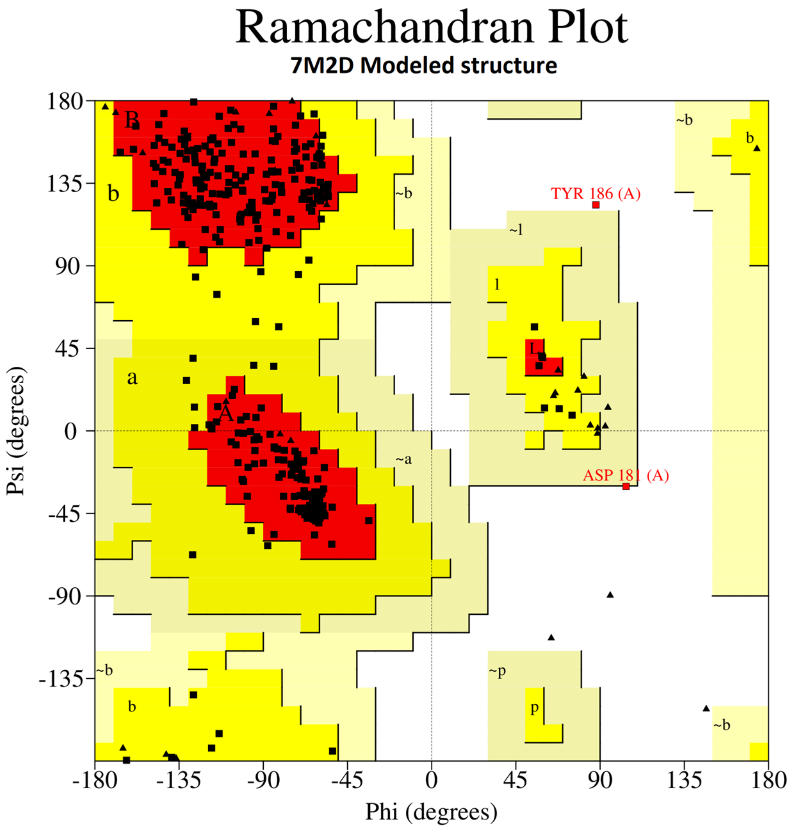

Figure S2. Ramachandran plot of the modeled EBOV-GP (7M2D) structure showing the distribution of backbone dihedral angles.

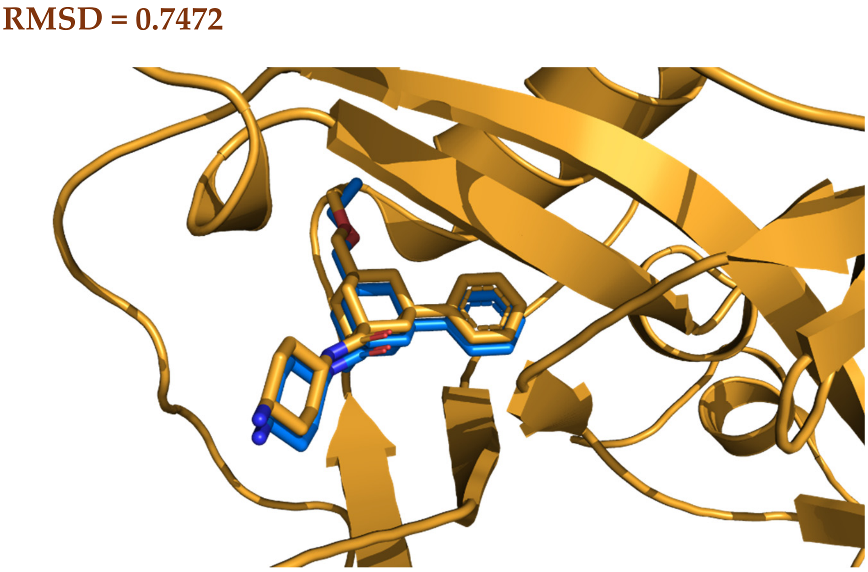

Figure S3. Best predicted pose of co-crystallized ligand (light-orange) superimposed with X-ray (bleu) inside EBOV-GP.

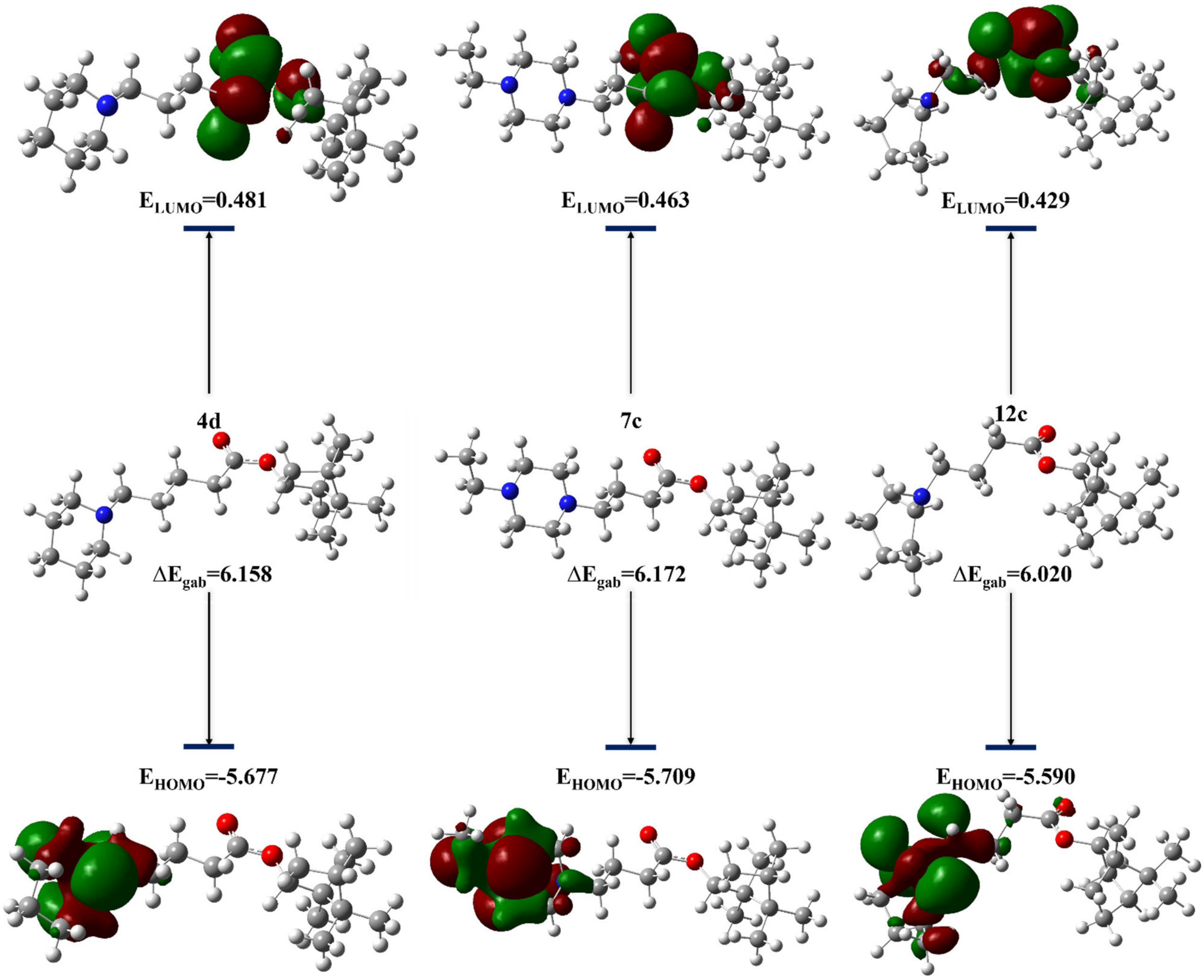

Figure S4. Optimized structures and frontier molecular orbitals (HOMO-LUMO) of the three lead compounds.

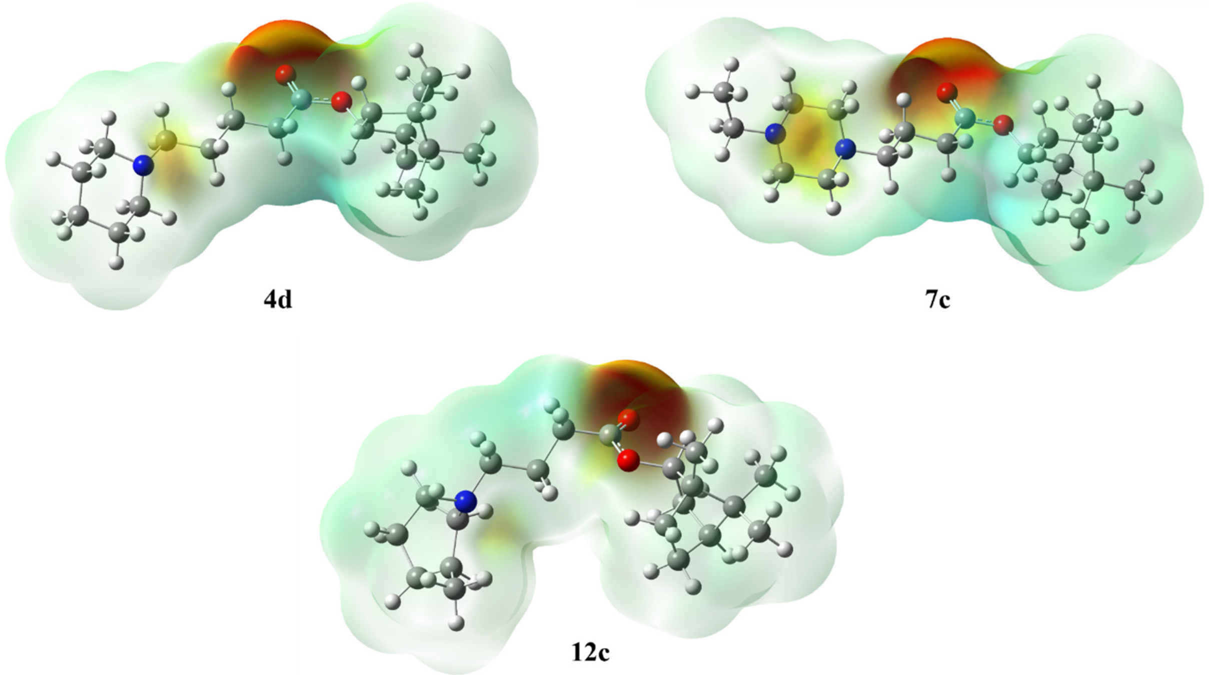

Figure S5. MEP analyses of the three lead compounds.

Table S1: Chemical structure of the 56 terpenoids derivatives and their corresponding IC_{50} and pIC_{50}

Names	Structures	pIC_{50}	IC_{50} (μM)
**(1R,2R,4R)-23**	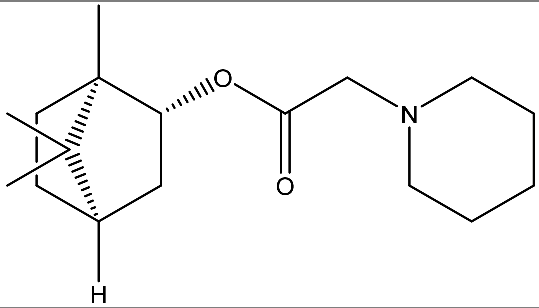	5.54	2.9
**(1R,2R,4R)-24**	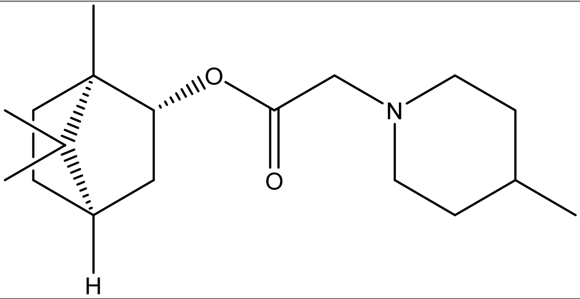	5.51	3.1
**(1S,2S,4S)-23**	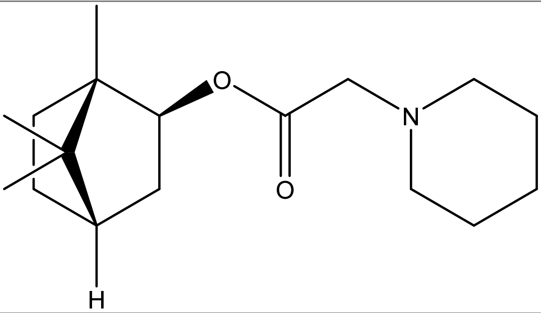	5.44	3.6
**(1S,2S,4S)-24**	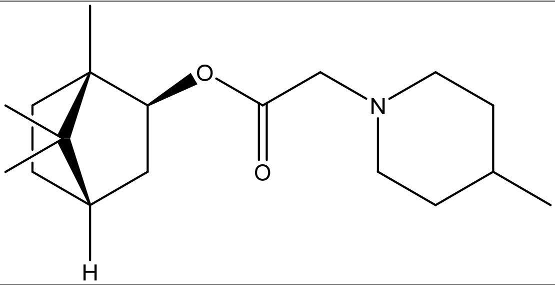	5.36	4.4
**10a**	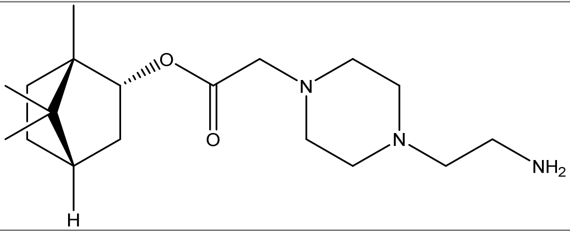	5.66	2.2
**10c**	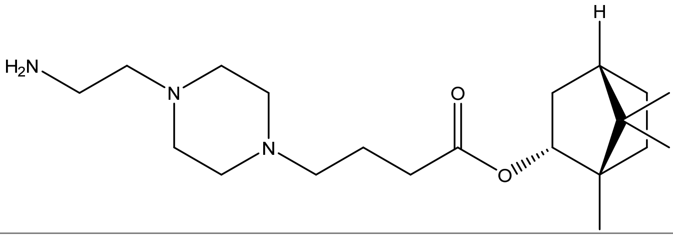	6.22	0.6

**11a**	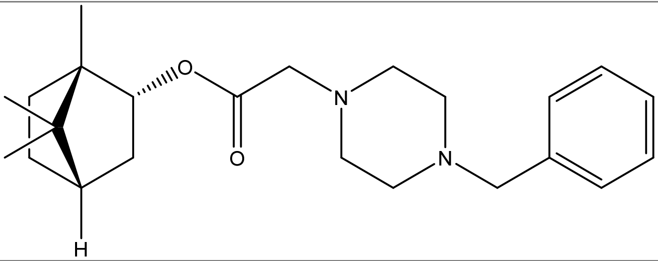	5.89	1.3
**11b**	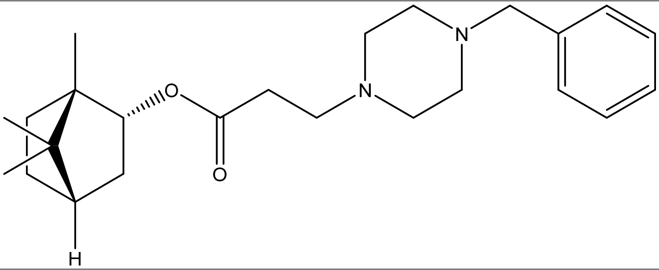	6.52	0.3
**11c**	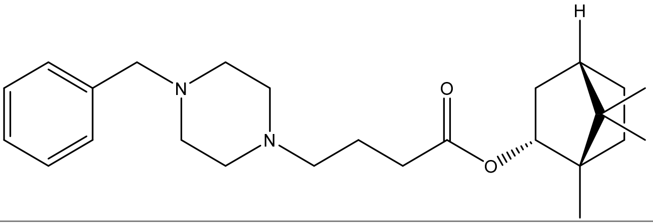	6.52	0.3
**12a**	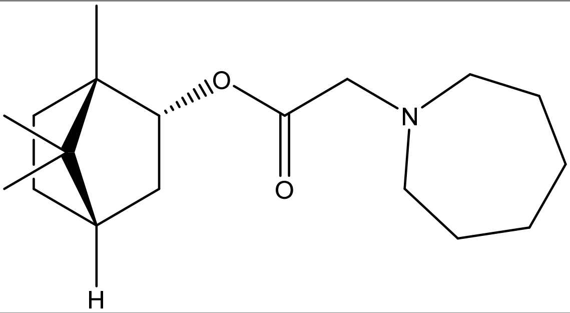	6.00	1
**12b**	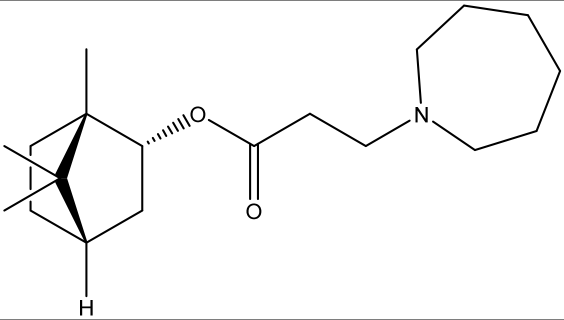	5.29	5.1
**12c**	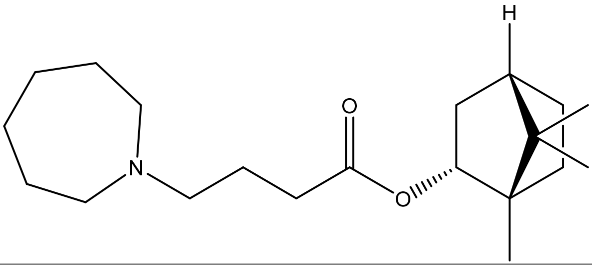	6.70	0.2

**13a**	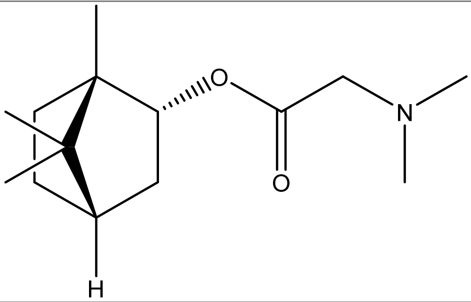	3.60	250.7
**13b**	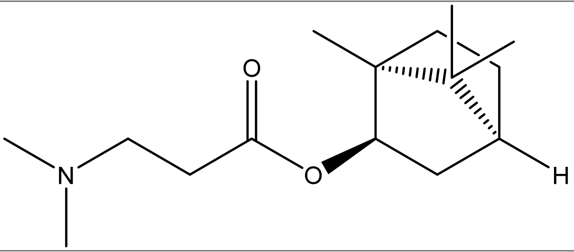	4.52	30
**13c**	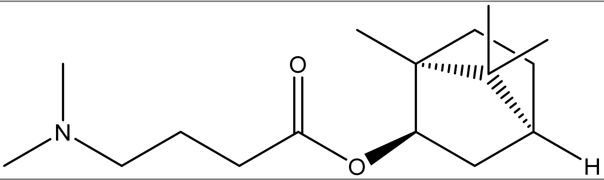	5.82	1.5
**14a**	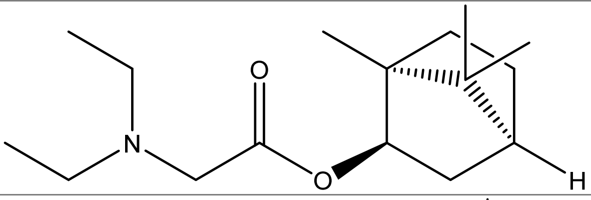	5.25	5.6
**14b**	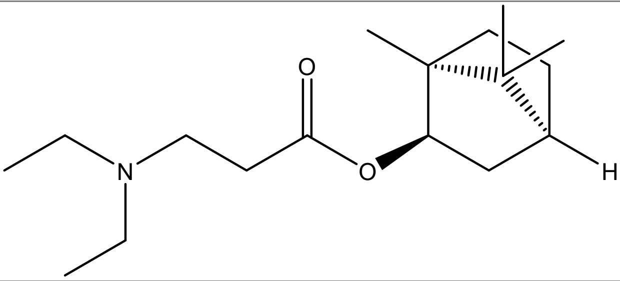	4.91	12.4
**14c**	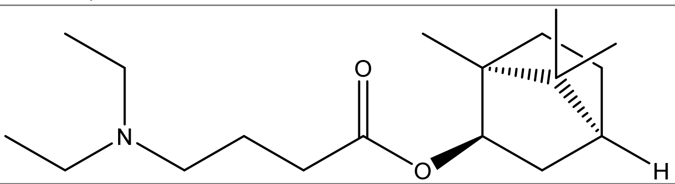	6.52	0.3
**18a**	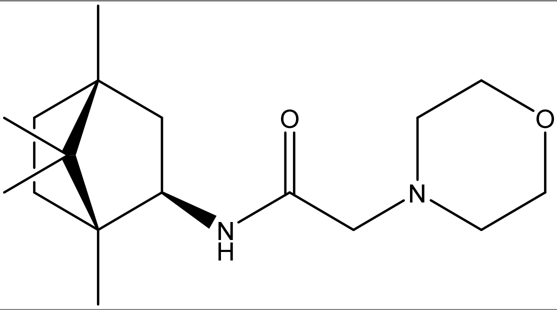	5.24	5.7

**18b**	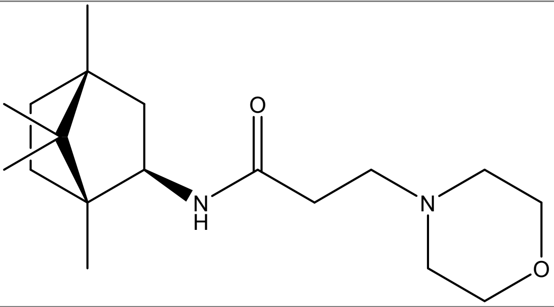	4.66	22.1
**18c**	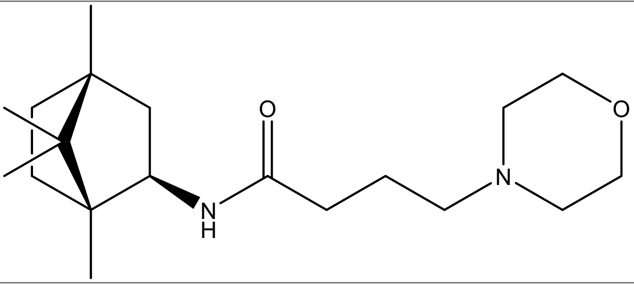	5.03	9.4
**19a**	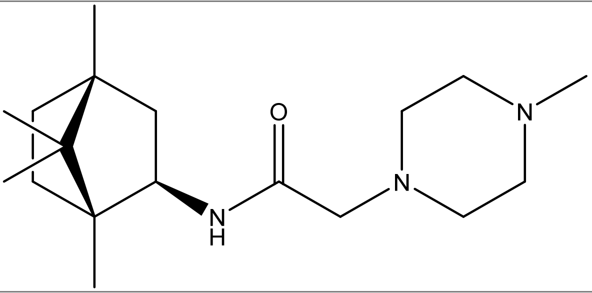	4.88	13.3
**19b**	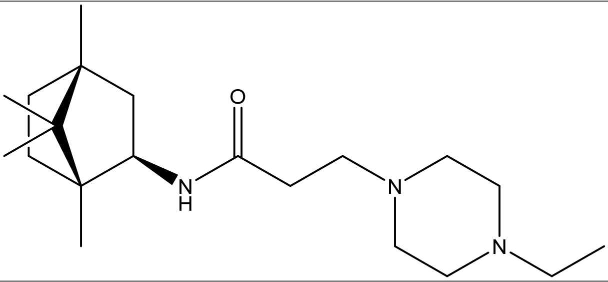	5.59	2.6
**19c**	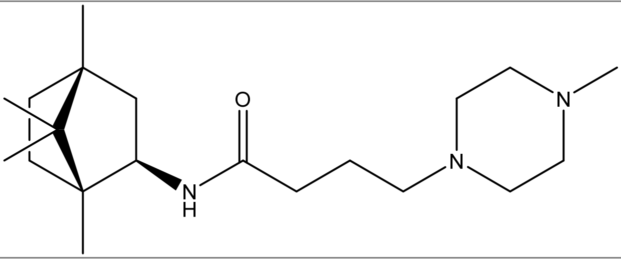	5.60	2.5
**20a**	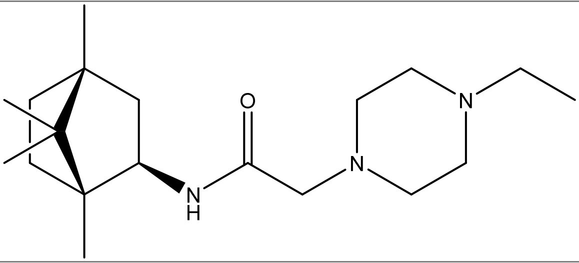	5.07	8.5

**20b**	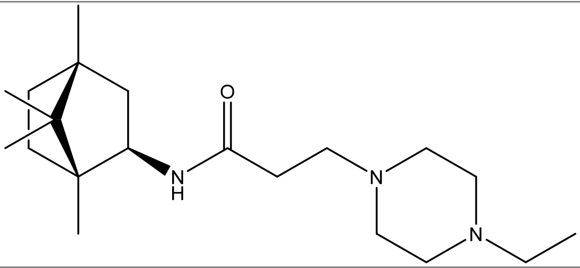	6.22	0.6
**20c**	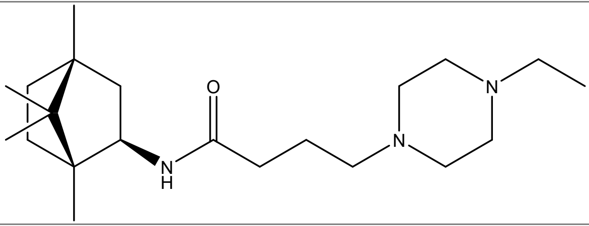	5.68	2.1
**21a**	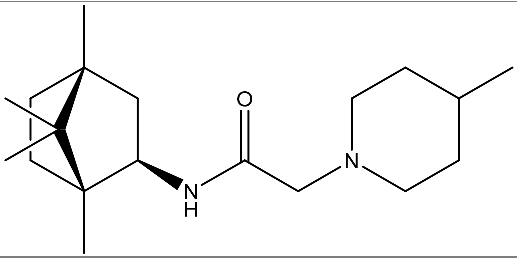	4.57	27
**21b**	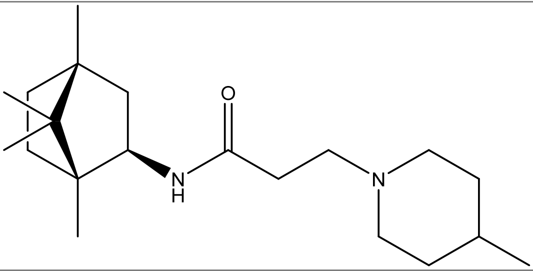	5.59	2.6
**21c**	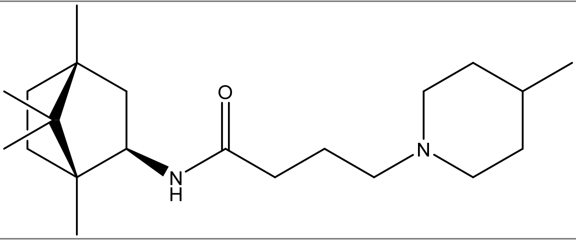	5.47	3.4
**3a**	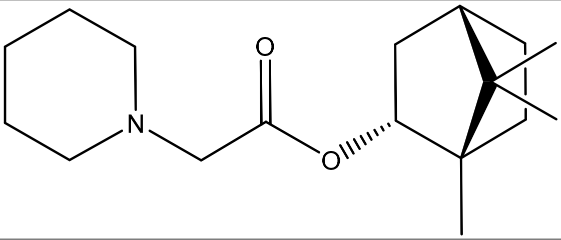	4.80	15.7
**3b**	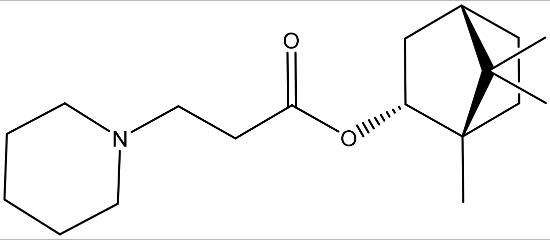	5.77	1.7

**3b_HCl**	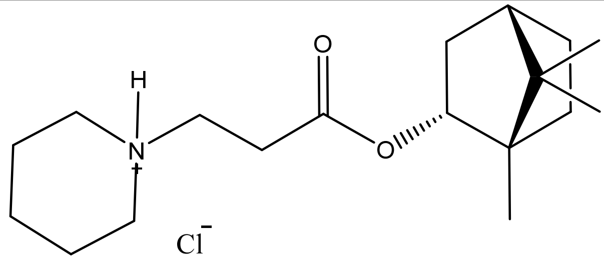	6.10	0.8
**3c**	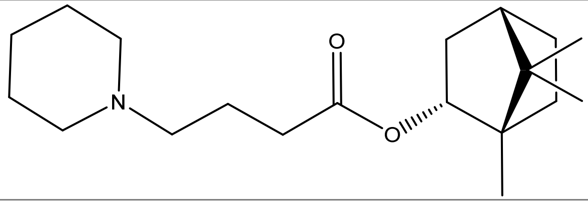	6.22	0.6
**3d**	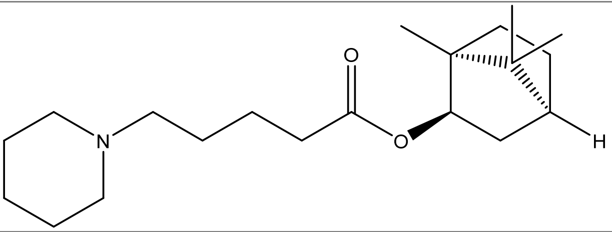	6.52	0.3
**4a**	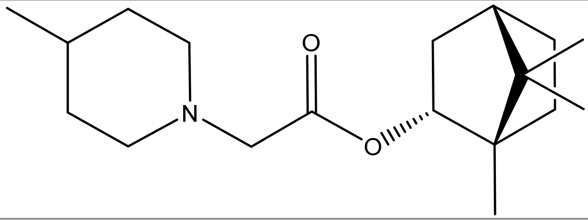	5.07	8.5
**4b**	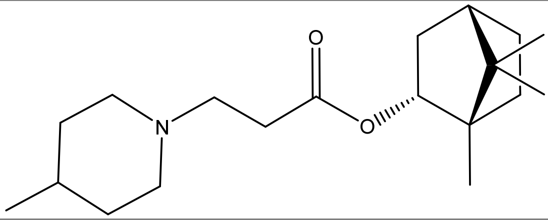	6.52	0.3
**4b_HCl**	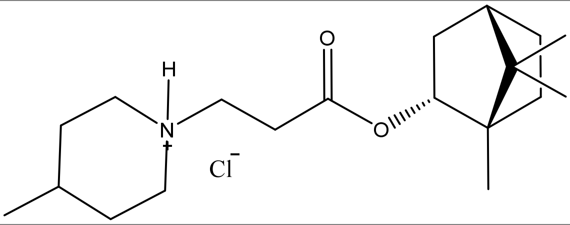	6.40	0.4
**4c**	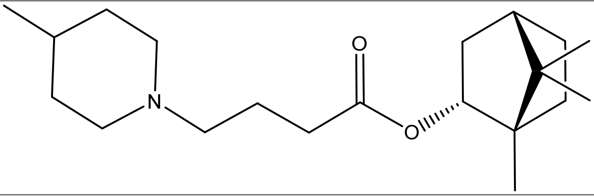	5.03	9.3
**4d**	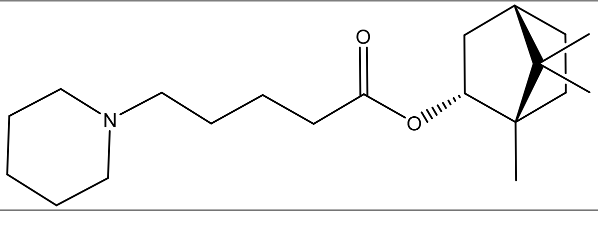	6.70	0.2

**5a**	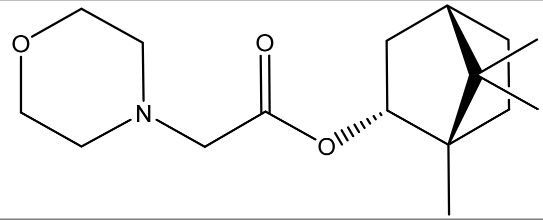	4.60	24.9
**5b**	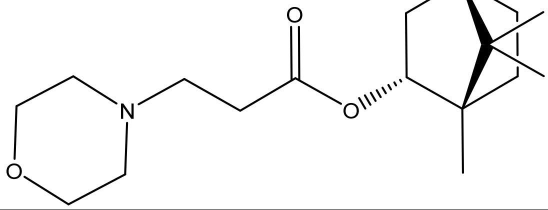	5.07	8.5
**5c**	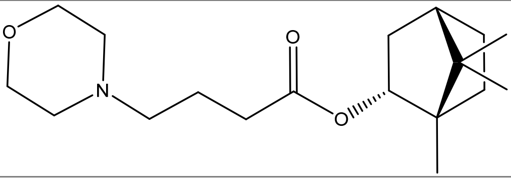	5.19	6.5
**6a**	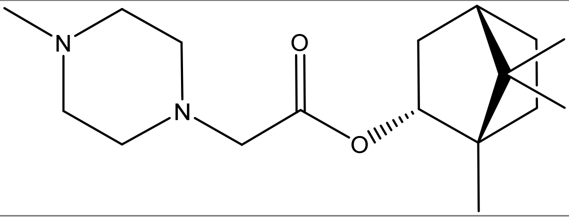	5.17	6.8
**6b**	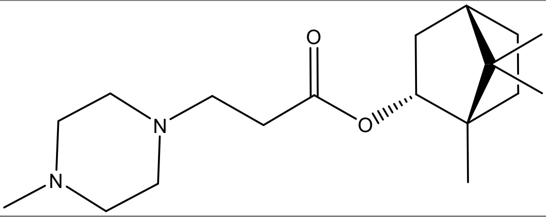	6.52	0.3
**6c**	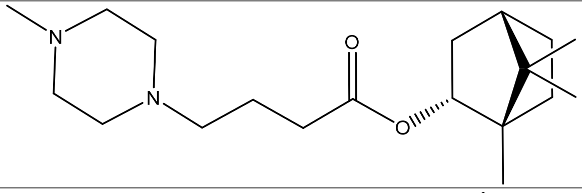	6.22	0.6
**7a**	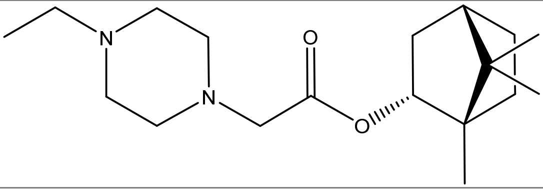	5.80	1.6
**7b**	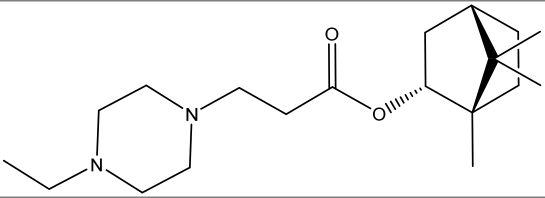	5.80	1.6
**7c**	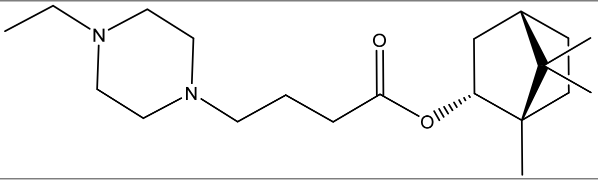	7.00	0.1

**8a**	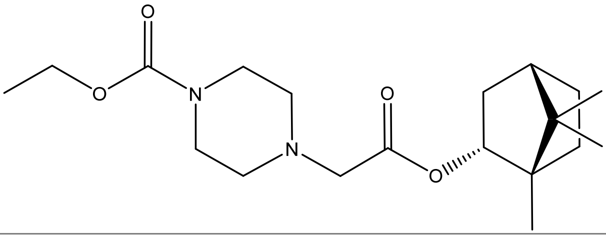	4.70	19.9
**8b**	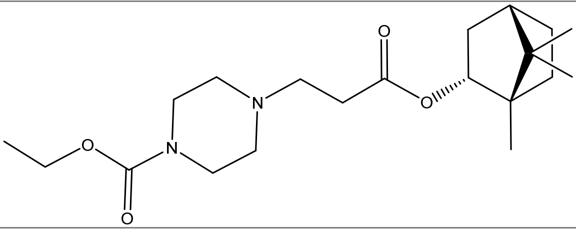	4.55	28.3
**8c**	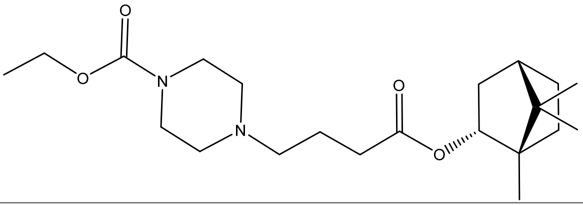	5.59	2.6
**9a**	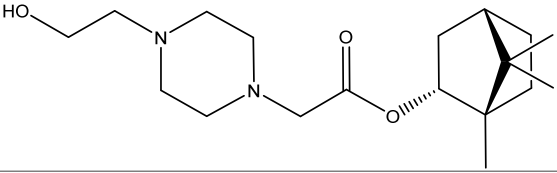	4.86	13.9
**9b**	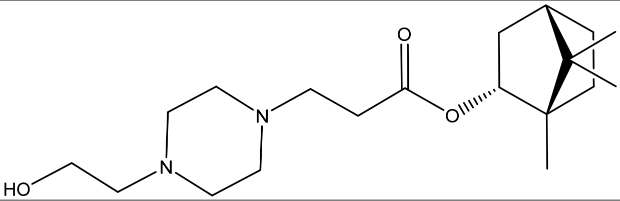	5.74	1.8
**9c**	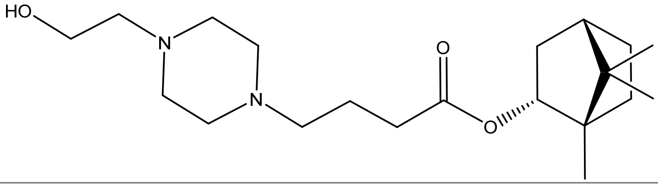	6.52	0.3

Table S2: Molecular descriptors and their description.

**Descriptor**	**Explanation**
**VE3sign_X**	Logarithmic coefficient sum of the last eigenvector from chi matrix
**VE3sign_Dt**	Logarithmic coefficient sum of the last eigenvector from detour matrix
**SpMAD_AEA(dm)**	Spectral mean absolute deviation from augmented edge adjacency mat. weighted by dipole moment
**MaxssCH2**	Maximum ssCH2

Table S3. Molecular docking interactions of the top eleven newly designed compounds, lead compounds (7c. 12c. and 4d), and toremifene within the active site of EBOV-GP.

Compound	Category	Type	Residues	Distance
**L-60**	Hydrogen Bond	Conventional HB	A:ARG64	3.0533
	Hydrogen Bond	Conventional HB	A:ARG64	1.9369
	Hydrogen Bond	Conventional HB	A:PHE193	1.8086
	Hydrophobic	Alkyl	A:LEU184	5.2912
	Hydrophobic	Pi-Alkyl	A:PHE194	5.1168
**L-3796**	Hydrogen Bond	Conventional HB	A:ARG64	1.9783
	Hydrophobic	Pi-Sigma	A:VAL66	3.8248
	Hydrophobic	Pi-Sigma	B:LEU515	3.5052
	Hydrophobic	Alkyl	A:VAL66	3.8517
	Hydrophobic	Alkyl	A:LEU68	3.7710
	Hydrophobic	Alkyl	B:LEU515	4.8806
	Hydrophobic	Alkyl	B:LEU558	3.1710
	Hydrophobic	Pi-Alkyl	A:ALA101	4.5387
**M-1074**	Hydrophobic	Alkyl	A:VAL66	3.6689
	Hydrophobic	Alkyl	A:LEU68	4.8367
	Hydrophobic	Alkyl	A:ALA101	4.7994
	Hydrophobic	Alkyl	B:LEU515	4.5709
	Hydrophobic	Alkyl	B:MET548	4.9303
	Hydrophobic	Alkyl	B:LEU554	5.1668
	Hydrophobic	Pi-Alkyl	B:TYR517	3.7508
**M-1618**	Hydrogen Bond	Conventional HB	A:PHE193	1.9802
	Hydrophobic	Alkyl	A:VAL66	3.6915
	Hydrophobic	Alkyl	A:LEU68	4.8908
	Hydrophobic	Alkyl	A:ALA101	4.9667
	Hydrophobic	Alkyl	B:LEU515	4.4073
	Hydrophobic	Alkyl	B:LEU554	4.8492
	Hydrophobic	Alkyl	B:LEU558	5.3971
	Hydrophobic	Alkyl	B:MET548	5.0860
	Hydrophobic	Pi-Alkyl	B:TYR517	4.6634
**L-874**	Hydrophobic	Alkyl	A:VAL66	3.6382
	Hydrophobic	Alkyl	A:LEU68	5.1774
	Hydrophobic	Alkyl	A:ALA101	5.0298
	Hydrophobic	Alkyl	A:LEU184	5.0015
	Hydrophobic	Alkyl	A:LEU186	5.4231
	Hydrophobic	Alkyl	B:LEU515	4.4316
	Hydrophobic	Alkyl	B:LEU558	5.4197
**L-1366**	Hydrogen Bond	Conventional HB	A:PHE193	2.5125
	Hydrophobic	Alkyl	A:VAL66	3.6382
	Hydrophobic	Alkyl	A:LEU68	5.1830
	Hydrophobic	Alkyl	A:ALA101	5.0237
	Hydrophobic	Alkyl	A:LEU184	5.0184
	Hydrophobic	Alkyl	A:LEU186	5.3890

	Hydrophobic	Alkyl	B:LEU515	4.4340
	Hydrophobic	Alkyl	B:LEU558	5.4255
**M-1205**	Hydrophobic	Pi-Sigma	A:VAL66	3.6628
	Hydrophobic	Pi-Sigma	B:LEU515	3.3434
	Hydrophobic	Alkyl	B:LEU554	5.1624
	Hydrophobic	Alkyl	B:LEU558	5.3741
	Hydrophobic	Pi-Alkyl	A:ALA101	4.8872
**M-1435**	Hydrophobic	Pi-Sigma	A:VAL66	3.7236
	Hydrophobic	Pi-Sigma	B:LEU515	3.4307
	Hydrophobic	Alkyl	A:ALA101	3.8016
	Hydrophobic	Alkyl	B:LEU554	4.9792
	Hydrophobic	Pi-Alkyl	B:TYR517	4.2162
	Hydrophobic	Pi-Alkyl	A:LEU68	5.3979
	Hydrophobic	Pi-Alkyl	A:ALA101	4.7458
**L-832**	Hydrogen Bond	Conventional HB	A:ARG136	2.9858
	Hydrophobic	Alkyl	A:ARG136	5.4268
**L-1512**	Hydrophobic	Alkyl	A:VAL66	3.6947
	Hydrophobic	Alkyl	A:ALA101	4.7780
	Hydrophobic	Alkyl	B:LEU515	4.5354
	Hydrophobic	Alkyl	B:MET548	5.4214
	Hydrophobic	Alkyl	B:LEU554	4.816
**L-1542**	Hydrophobic	Pi-Sigma	B:TYR517	3.9809
	Hydrophobic	Alkyl	A:VAL66	3.7051
	Hydrophobic	Alkyl	A:LEU68	5.0353
	Hydrophobic	Alkyl	A:ALA101	4.8849
	Hydrophobic	Alkyl	B:LEU515	4.4193
	Hydrophobic	Alkyl	B:MET548	5.4695
	Hydrophobic	Alkyl	B:LEU554	4.8096
	Hydrophobic	Alkyl	B:LEU558	5.4113
**7c**	Hydrogen Bond	Carbon HB	A:ASN61	3.7415
	Hydrophobic	Alkyl	A:VAL66	5.0060
	Hydrophobic	Alkyl	A:ALA101	4.8541
	Hydrophobic	Pi-Alkyl	B:TYR517	4.7275
**12c**	Hydrophobic	Pi-Sigma	B:TYR517	3.8750
	Hydrophobic	Alkyl	A:ARG64	5.3039
	Hydrophobic	Alkyl	A:LEU184	4.9389
	Hydrophobic	Alkyl	B:LEU554	4.7938
	Hydrophobic	Alkyl	B:LEU558	4.7523
**4d**	Hydrophobic	Alkyl	A:VAL66	3.6140
	Hydrophobic	Alkyl	A:LEU68	4.9508
	Hydrophobic	Alkyl	A:ALA101	5.0075
	Hydrophobic	Alkyl	B:LEU515	4.4379
	Hydrophobic	Alkyl	B:LEU554	5.1841
**TOR**	Electrostatic	Pi-Cation	A:ARG64	3.4961
	Electrostatic	Pi-Cation	A:ARG64	3.8333
	Hydrophobic	Pi-Pi T-shaped	A:PHE194	4.5219

	Hydrophobic	Pi-Alkyl	A:PHE194	4.8524
	Hydrophobic	Pi-Alkyl	A:ARG64	4.7189
	Hydrophobic	Pi-Alkyl	A:ALA101	4.7541
